# Commensal-derived short-chain fatty acids disrupt lipid membrane homeostasis in *Staphylococcus aureus*

**DOI:** 10.1128/mbio.01392-25

**Published:** 2025-11-28

**Authors:** Joshua R. Fletcher, Lisa A. Hansen, Julia R. Hoyser, Allison E. Hanna, Richard Martinez, Christian D. Freeman, Niall T. Thorns, Mitchell R. Penningroth, Alex R. Villarreal, Grace A. Vogt, Matthew A. Tyler, Kelly M. Hines, Ryan C. Hunter

**Affiliations:** 1 Department of Microbiology & Immunology, University of Minnesota 5635, Minneapolis, Minnesota, USA; 2 Department of Population Health and Pathobiology, North Carolina State University College of Veterinary Medicine 70727, Raleigh, North Carolina, USA; 3 Department of Microbiology & Immunology, Jacobs School of Medicine and Biomedical Sciences, University at Buffalo 12292 , Buffalo, New York, USA; 4 Department of Chemistry, University of Georgia 1355, Athens, Georgia, USA; 5 Department of Otolaryngology, University of Minnesota 5635, Minneapolis, Minnesota, USA; Universite de Geneve, Geneva, Switzerland

**Keywords:** *Staphylococcus aureus*, anaerobes, short-chain fatty acids, lipidomics, branched-chain fatty acids

## Abstract

The role of commensal anaerobic bacteria in chronic respiratory infections is unclear, yet they can exist in abundances comparable to canonical pathogens *in vivo*. Their contributions to the metabolic landscape of the host environment may influence pathogen behavior by competing for nutrients and creating inhospitable conditions via toxic metabolites. Here, we show that the anaerobe-derived short-chain fatty acids (SCFAs) propionate and butyrate negatively affect *Staphylococcus aureus* physiology by disrupting branched-chain fatty acid (BCFA) metabolism. In turn, alterations to BCFA abundance impair *S. aureus* growth, compromise membrane integrity, diminish expression of the accessory gene regulator quorum-sensing system, and increase sensitivity to membrane-targeting antimicrobials. Disrupted BCFA metabolism also reduced *S. aureus* fitness in competition with *Pseudomonas aeruginosa*, suggesting that airway microbiome composition and the metabolites they exchange can directly impact pathogen succession over time. The pleiotropic effects of SCFAs on *S. aureus* fitness and their ubiquity as metabolites in the human host also suggest that they may be effective as adjuvants to traditional antimicrobial agents when used in combination.

**IMPORTANCE:**

*Staphylococcus aureus* is a primary pathogen of chronic airway disease yet is also found in the upper airways of 30%–50% of the population to no obvious detriment. Thus, identifying the host and/or microbial factors that tip the balance between its commensal and pathogenic states may be key to its control. Here, we reveal that short-chain fatty acids produced by commensal microbiota promote a marked remodeling of the *S. aureus* lipid membrane that, in turn, sensitizes the pathogen to antimicrobials, disrupts accessory gene regulator quorum signaling, and reduces its competitive fitness. Altogether, these data suggest that co-colonizing microbiota and the metabolites they exchange with *S. aureus* may be key players in the microbial ecology of airway disease.

## INTRODUCTION

*Staphylococcus aureus* is a Gram-positive pathogen often found in polymicrobial infections of the cystic fibrosis (CF) lung and upper airways of individuals with chronic rhinosinusitis (CRS) (1–5). Despite its arsenal of virulence factors and association with respiratory infections, *S. aureus* is also commonly present in the airways of healthy individuals to no obvious detriment (6). Since *S. aureus* can be both a pathogen and commensal, understanding how it integrates environmental cues to regulate its metabolism and virulence is essential.

A common feature of both CF and CRS is the accumulation of viscous airway mucus, resulting in hypoxic microenvironments and colonization by anaerobic bacteria that can utilize mucin glycoproteins as growth substrates (5, 7–9). As a result, anaerobes generate short-chain fatty acids (SCFAs) that stimulate host inflammation, serve as carbon sources for pathogens like *Pseudomonas aeruginosa*, or, in the case of propionate and butyrate, impair *S. aureus* growth (7, 10–12).

Mechanisms underlying SCFA-mediated inhibition of *S. aureus* are unknown, but emerging evidence implicates lipid metabolism and cell wall stress (11, 13). For instance, perturbation of teichoic acids renders *S. aureus* susceptible to inhibition by propionate *in vitro* and in a murine wound model (13). Moreover, FadX (putatively involved in fatty acid degradation) is required for optimal *S. aureus* growth in propionate, while a *codY* mutant grows significantly better than wild type in butyrate (11). CodY is a master regulator of metabolism and virulence, and among the most highly expressed genes in a *codY* mutant are involved in branched-chain amino acid (BCAA) biosynthesis (14, 15). BCAAs (isoleucine, leucine, valine) are substrates for production of branched-chain fatty acids (BCFAs), which are highly abundant in the *S. aureus* membrane. The branched- to straight-chain fatty acid ratio is essential for regulating membrane fluidity as environmental conditions change (16–20). Recently, a *codY* mutant was shown to have elevated anteiso BCFAs in its membrane, and the activity of the Sae two-component system was sensitive to their presence (21). Disruption of BCFA production via mutation of branched-chain ketoacid dehydrogenase (Bkd) results in poor growth, increased membrane rigidity, and sensitivity to environmental stresses (17–19).

Given these observations, we hypothesized that propionate and butyrate disrupt *S. aureus* lipid membrane homeostasis by decreasing BCFA abundance. We found that isoleucine improved *S. aureus* growth in the presence of propionate and butyrate, whereas leucine and valine worsened it. Mutants defective in anteiso-BCFA synthesis were hypersensitive and not rescued by isoleucine. Targeted lipidomics revealed that *S. aureus* grown in propionate- and butyrate-supplemented media had decreased BCFA to straight-chain FA ratios, with an accompanying decrease in membrane polarization, increased susceptibility to membrane-targeting antimicrobials, reduced accessory gene regulator (agr) signaling, and diminished competitiveness against *P. aeruginosa. S. aureus* clinical isolates behaved similarly across phenotypic assays, indicating that SCFAs act on conserved molecular targets.

## RESULTS

### Propionate and butyrate alter the *S. aureus* transcriptome and proteome

Airway microbiota consist of complex communities, with several taxa originating from the oral cavity (22–24). In CF sputum and CRS mucus, *Streptococcus*, *Veillonella, Prevotella*, and *Fusobacterium* spp. are prevalent and consistently abundant, yet the contributions of these strict and facultative anaerobes to disease remain poorly defined (3–5, 25). Our previous work showed spent supernatants from *Fusobacterium nucleatum* impair *S. aureus* growth, an effect attributed to the SCFAs propionate and butyrate (11). Here, our goal was to further dissect the effects of commensal-derived SCFAs on *S. aureus* to identify their mechanisms of action.

**Fig 1:**
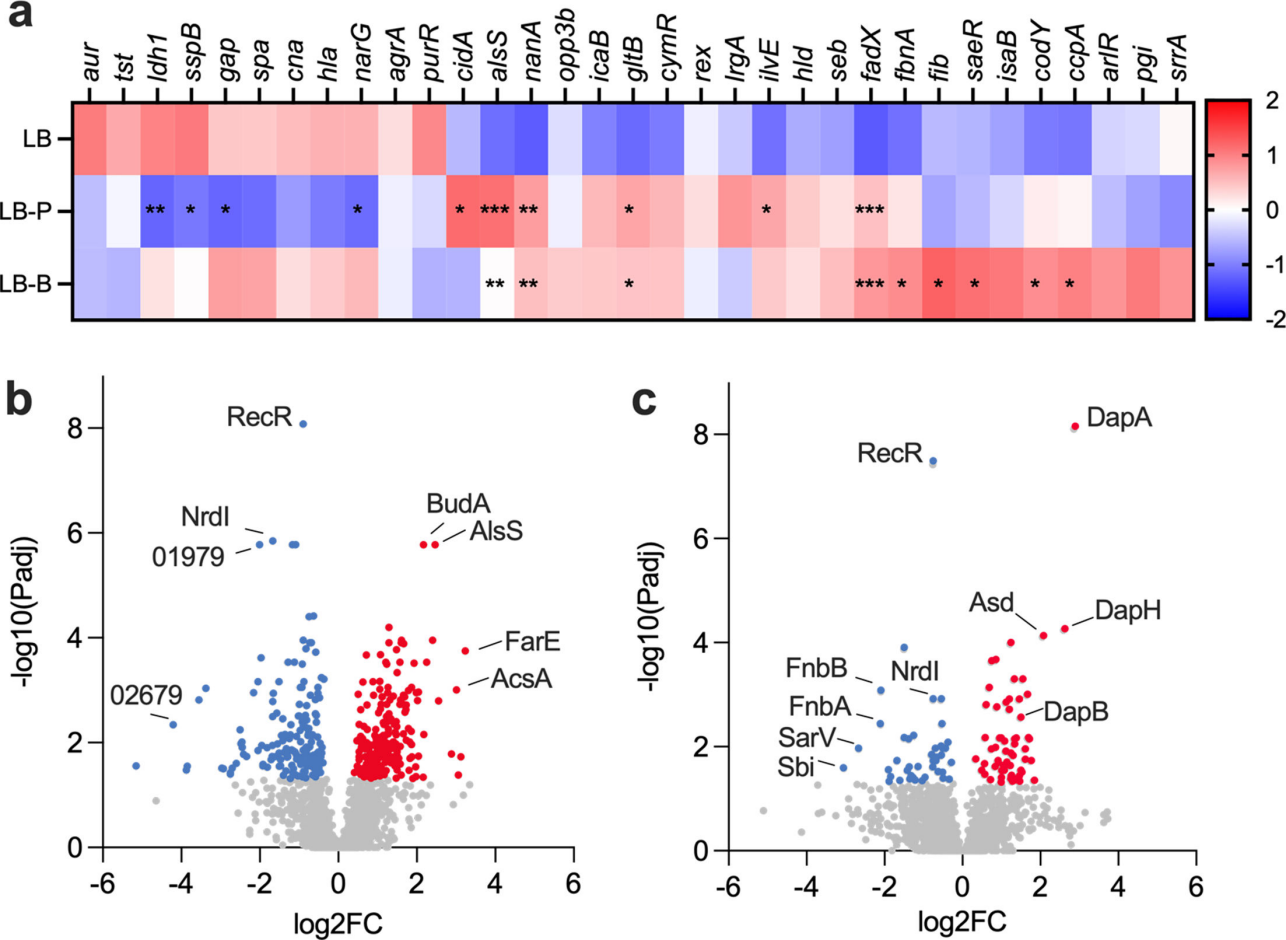
Propionate and butyrate alter *S. aureus* transcript and protein abundances. (**a**) Heatmap depicting Z-scores of log_10_-transformed NanoString counts from *S. aureus* JE2 grown to OD_600_ of ~0.2–0.3 in LB with or without 100 mM of sodium propionate or sodium butyrate (*n* = 3 per condition). Transcripts that exhibited greater than twofold change in abundance relative to growth in LB were considered statistically significant at a Benjamini-Hochberg-adjusted *P*-value ≤0.05 (***<0.001, **<0.01, *<0.05). (**b and c**) Volcano plots of the *S. aureus* proteome in LB supplemented with propionate (**b**) or butyrate (**c**) compared to LB alone. Complete NanoString and proteomics data output can be found in Data sets S1 and S2, respectively.

**Fig 2:**
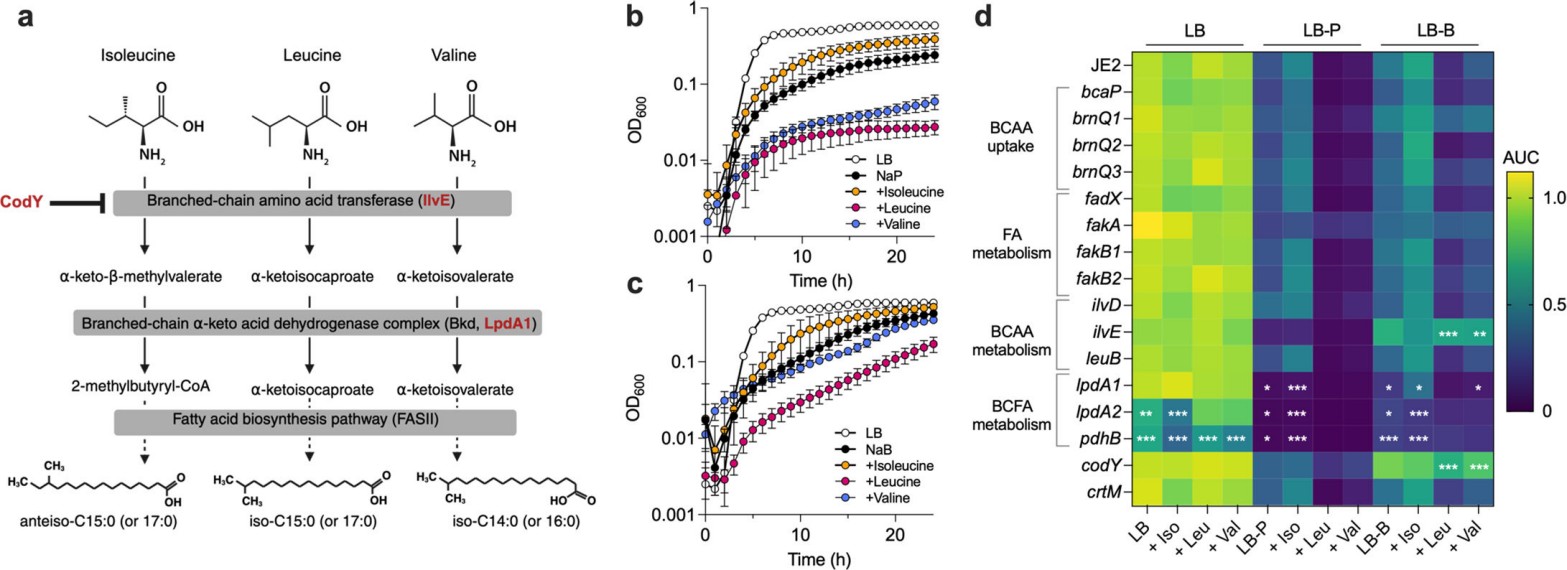
Isoleucine supplementation partially relieves growth inhibition by propionate and butyrate, while leucine and valine enhance it. (**a**) Graphical depiction of the conversion of BCAAs to α-ketoacids by IlvE, then to CoA esters by the branched-chain ketoacid dehydrogenase (BKD) for their subsequent use as substrates for FASII. Adapted from Chan and Wiedmann (29). The BKD complex includes LpdA1. (**b and c**) Growth curves of *S. aureus* JE2 in LB with 100 mM of (**b**) sodium propionate (NaP) or (**c**) sodium butyrate (NaB), supplemented with 1 mg/mL of the indicated branched-chain amino acid. Error bars represent standard error of the mean (*n* = 3 biological replicates) for each time point. (**d**) Heatmap depicting the normalized area under the curve (AUC) of *S. aureus* JE2 and transposon mutants in genes associated with BCAA uptake/metabolism or fatty acid metabolism (*n* = 6 growth curves per strain, per condition). Data were normalized to JE2 grown in LB alone and are presented as percent area under the curve. Large normalized AUC indicates robust growth and is depicted in lighter green to yellow, while low values indicate poor growth and are shown in darker green to dark blue. Statistical significance of each mutant compared to JE2 under a given growth condition was tested using an ordinary two-way ANOVA (*, *P* < 0.05; **, *P* < 0.01; ***, *P* < 0.001). Statistical comparisons were too numerous to depict graphically and are shown in Data set S3. Additional growth curve data are shown in Fig. S3.

We first used a custom NanoString codeset targeting 33 transcriptional regulators, virulence factors, and metabolic genes to gain preliminary insight into differential gene expression in *S. aureus* JE2 when grown in LB supplemented with either propionate or butyrate relative to LB alone (Fig. 1a; Data set S1). Since the roles of these genes in *S. aureus* physiology are relatively well described, we reasoned that any alterations in their expression would help identify downstream targets for further investigation. Each SCFA induced a distinct expression profile, with 10 (propionate) and 9 (butyrate) transcripts reaching significance versus LB-grown controls (*P*_adj_ <0.05). Four transcripts decreased in propionate, whereas all nine in butyrate increased. Several were previously identified in *S. aureus* grown in *F. nucleatum* supernatants (11), despite different basal media, underscoring the specific effect of SCFAs on *S. aureus* physiology. Although propionate and butyrate elicited distinct responses, some commonalities were also observed. For instance, expression of *alsS*, *nanA*, *gltB*, and *fadX* increased in LB medium containing either SCFA relative to LB alone. *alsS* is annotated as an acetolactate synthase, while *nanA*, *gltB*, and *fadX* are involved in sialic acid, glutamate, and fatty acid metabolism, respectively (26, 27). Additionally, expression of *ilvE*, which encodes a CodY-regulated BCAA aminotransferase involved in anteiso-BCFA synthesis (28) (Fig. 2a), increased in both SCFA conditions but reached statistical significance only in propionate. Since BCFAs are essential to *S. aureus*, we posit that *ilvE* expression is largely constitutive, potentially limiting the magnitude of its SCFA-induced change (18, 21).

To complement our transcriptomic data, we profiled the *S. aureus* proteome under identical experimental conditions (Fig. 1b and c; Data set S2). Compared to growth in LB alone, 370 (propionate) and 103 (butyrate) proteins were differentially abundant (*P* < 0.05), with 80 shared between conditions (Fig. S1). RecR was significantly lower when either SCFA was present, as was the NrdI ribonucleotide reductase stimulatory protein. Acetolactate synthase proteins BudA and AlsS were highly induced by propionate only. Conversely, the dihydropicolinate synthases DapA, DapB, and DapH were significantly higher in butyrate only, suggesting cell wall remodeling. N-acetylneuraminate lyase (NanA) was induced by propionate, consistent with transcript levels. In contrast, FbnA was lower in both SCFAs compared to LB alone, despite showing significant transcriptional induction in butyrate. This suggests a disconnect between *fbnA* transcription and translation. Likewise, CodY protein abundance was consistent across media, despite *codY* transcripts being modestly induced by both SCFAs. IlvE was approximately twofold higher in both SCFA media but was not statistically significant.

Together, these transcriptomic and proteomic data reveal that propionate and butyrate elicit broad but distinct effects on *S. aureus*, converging on pathways linked to cell envelope homeostasis, metabolism, and virulence. Building on our previous findings linking SCFAs to lipid metabolism (11), we next focused on their impact on BCAAs and BCFAs.

### Isoleucine relieves SCFA-mediated growth inhibition

Given (i) increased *ilvE* expression during growth in SCFAs (Fig. 1a), (ii) robust growth of a *codY* mutant in butyrate (11), and (iii) elevated anteiso BCFAs in the *codY* mutant membrane (21), we hypothesized that SCFA-mediated growth inhibition was due to disruption of BCFA metabolism. If so, supplying exogenous BCAAs in excess of those in the base medium (Fig. S2) should mitigate this stress by providing additional BCFA precursors (Fig. 2a). To test this, JE2 was grown in LB supplemented with one BCAA (isoleucine, leucine, or valine; 1 mg/mL) either alone or in the presence of propionate or butyrate (Fig. 2b and c). Growth was impaired by both SCFAs alone but partially restored by isoleucine. Interestingly, growth was further impaired by leucine and valine, suggesting that iso-BCFAs derived from these substrates are detrimental when SCFAs are present. These data also align with previous studies showing that α-keto-β-methylvalerate (produced by deamination of isoleucine by IlvE) is the preferred substrate of Bkd (Fig. 2a), whereas leucine- and valine-derived intermediates are less efficiently utilized (20).

To further explore these phenotypes, we screened a series of *S. aureus* transposon mutants with putative roles in BCAA and BCFA metabolism (Fig. 2d; Table S1) for growth inhibition by propionate and butyrate, with and without excess BCAAs. Mutants were selected for their gene product’s involvement in BCAA uptake (*bcaP*, *brnQ1-3*), fatty acid metabolism (*fakA*, *fakB1*, *fakB2*, *fadX*), BCAA metabolism (*ilvD*, *ilvE*, *leuB*), or BCFA metabolism (*lpdA1*, *pdhB*). *lpdA2*, although not yet directly linked to BCFA metabolism, shares homology with *lpdA1* and lies downstream of *pdh* genes. A *codY* mutant was also included given its robust growth in butyrate relative to wild type (11). Finally, we included a staphyloxanthin mutant (*crtM*), as staphyloxanthin can influence membrane fluidity, which is diminished by reduced BCFA content (16, 17, 30, 31).

While several mutants displayed altered growth patterns relative to JE2, only *codY*::tn, *ilvE*::tn, *lpdA1*::tn, *lpdA2*::tn, and *pdhB*::tn reached significance in one or more media (Fig. 2d; Fig. S3, Data set S3). Despite the role of staphyloxanthin in regulating membrane fluidity, *crtM*::tn did not exhibit altered SCFA sensitivity. As expected, given our previous observations (11), *codY*::tn grew significantly better than the wild type in butyrate. Interestingly, *ilvE*::tn phenocopied *codY*::tn in butyrate but grew poorly in propionate and was not rescued by isoleucine. *lpdA1*::tn, *lpdA2*::tn, and *pdhB*::tn each grew poorly in propionate, and isoleucine supplementation likewise failed to rescue their growth. Growth of these three mutants in butyrate was higher than in propionate but still considerably worse than the parent JE2. *lpdA1*::tn exhibited isoleucine-enhanced growth in butyrate (though less than wildtype), while *lpdA2*::tn and *pdhB*::tn did not, showing that LpdA2 or another pathway may provide redundancy to *lpdA1*::tn when butyrate is present. Together, these data support our hypothesis that BCFA metabolism is disrupted by propionate and butyrate. However, IlvE’s paradoxical role, being essential for optimal growth in propionate but beneficially dispensable in butyrate, suggests a distinct, isoleucine-independent mechanism of butyrate tolerance.

### Propionate and butyrate disrupt membrane potential

**Fig 3:**
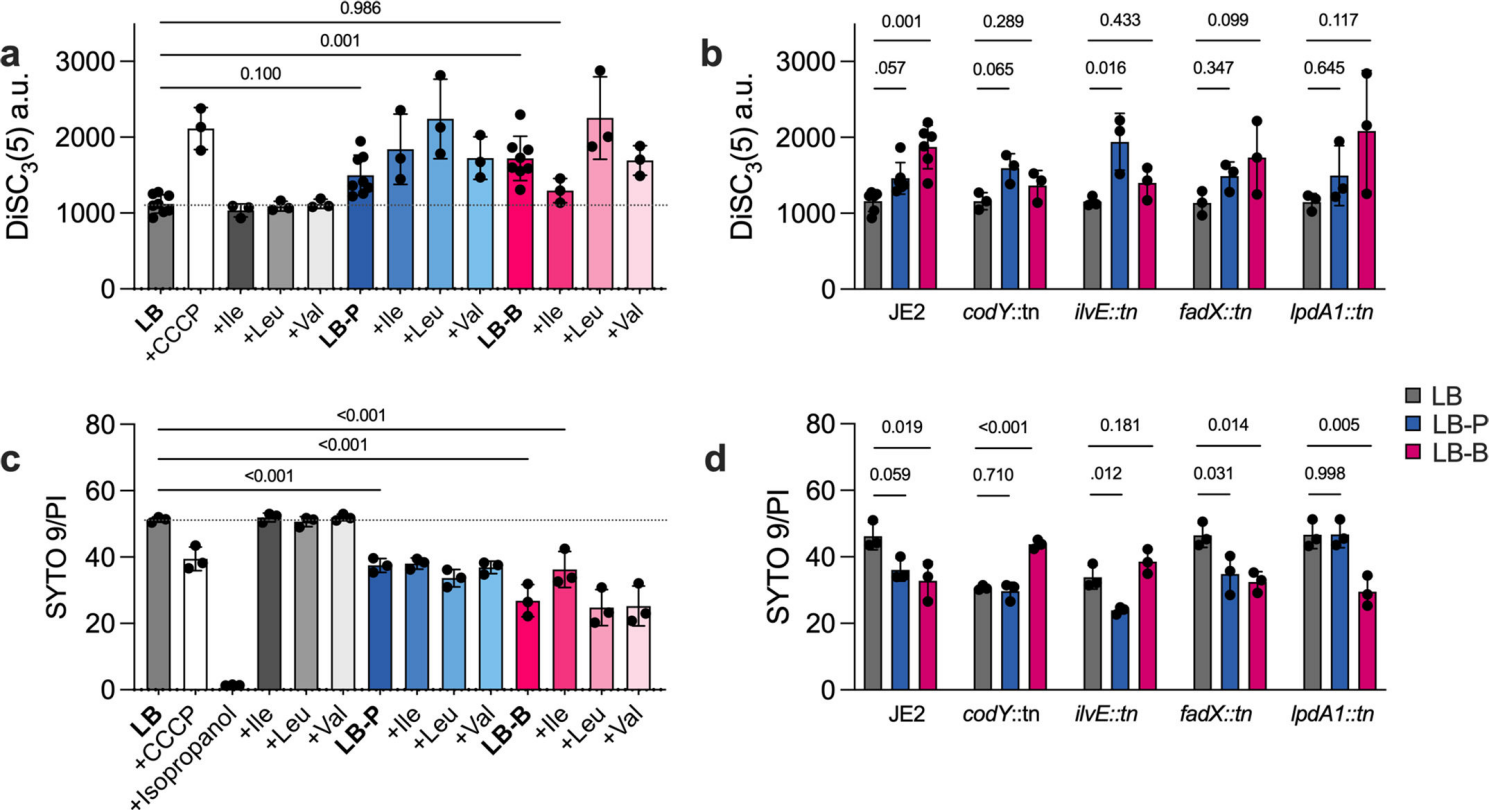
Propionate and butyrate disrupt *S. aureus* membrane potential. (**a**) Membrane potential of *S. aureus* JE2 grown in LB with or without 50 mM of sodium propionate or sodium butyrate (*n* = 9 per condition), with and without 1 mg/mL of branched-chain amino acids (*n* = 3), as measured by DiSC_3_(5) fluorescence. CCCP-treated cells grown in LB were included as a depolarization control (*n* = 3). Statistical significance of each condition was compared to LB alone (dashed line) using a one-way ANOVA with Dunnett’s multiple comparisons test. (**b**) JE2 and transposon mutants in genes associated with BCAA uptake/metabolism or fatty acid metabolism grown in LB ± SCFA (*n* = 3 per condition). For each mutant, statistical significance between each SCFA and LB alone was determined using a one-way ANOVA. (**c**) Membrane integrity of JE2 grown in LB with or without sodium propionate or sodium butyrate (*n* = 3), with and without BCAAs, as measured by LIVE/DEAD staining. (**d**) JE2 and transposon mutants in genes associated with BCAA uptake/metabolism or fatty acid metabolism grown in LB ± SCFA, as measured by LIVE/DEAD staining (*n* = 3 per condition). Complete statistical comparisons in each were too numerous to depict graphically and are shown in Data set S3.

We hypothesized that if SCFAs impair growth by reducing BCFA abundance, they would also compromise membrane integrity. To test this, we first measured membrane potential using 3,3′-dipropylthiacarbocyanine iodide (DiSC_3_(5)), a potentiometric fluorophore that accumulates in polarized membranes and self-quenches. Depolarization releases the dye, increasing fluorescence. Consistent with growth phenotypes in Fig. 2, JE2 grown in butyrate exhibited higher DiSC_3_(5) fluorescence relative to LB alone (*P* = 0.001), similar to depolarization induced by the protonophore carbonyl cyanide 3-chlorophenylhydrazone (CCCP) (*P* < 0.001) (Fig. 3a and b). Supplementation of LB + butyrate with isoleucine restored polarization to LB levels (*P* = 0.986). Propionate likewise increased fluorescence relative to LB, although this effect did not reach significance (*P* = 0.1).

Transposon mutants exhibited membrane potential patterns consistent with their growth phenotypes (Fig. 3b). *codY::tn* maintained polarization in butyrate (*P* = 0.289 versus *P* = 0.001 in wild-type JE2), while *ilvE::tn* reduced butyrate-induced depolarization (*P* = 0.277 versus *P* = 0.001) but exacerbated depolarization in propionate (*P* = 0.026 versus *P* = 0.031 in wild type), consistent with the paradoxical role of IlvE described above.

We also performed LIVE/DEAD staining, in which the propidium iodide (PI) “dead” stain is excluded from cells with intact chemiosmotic potential. In LB, JE2 exhibited a SYTO9 (“live”):PI ratio of ~51, which decreased following treatment with CCCP (*P* = 0.009) or isopropanol (*P* < 0.001). Growth in propionate (*P* = 0.002) and butyrate (*P* < 0.001) similarly decreased this ratio, consistent with membrane depolarization. Isoleucine supplementation partially restored the LB:butyrate ratio, but not to LB control levels. Transposon mutants again exhibited trends consistent with their growth phenotypes (Fig. 3d), mirroring DiSC_3_(5) fluorescence data. Together, these findings support that SCFAs impair *S. aureus* growth by compromising membrane integrity.

Finally, we used transmission electron microscopy to evaluate whether SCFAs alter cellular ultrastructure. Consistent with prior work linking altered BCFA abundance to increased peptidoglycan thickness (32), cells grown in propionate and butyrate exhibited increased cell wall dimensions (Fig. S4). However, aside from this change, no overt ultrastructural differences, including cytoplasmic membrane perturbation, were observed across conditions.

### SCFAs invert the branched- to straight-chain fatty acid ratio

**Fig 4:**
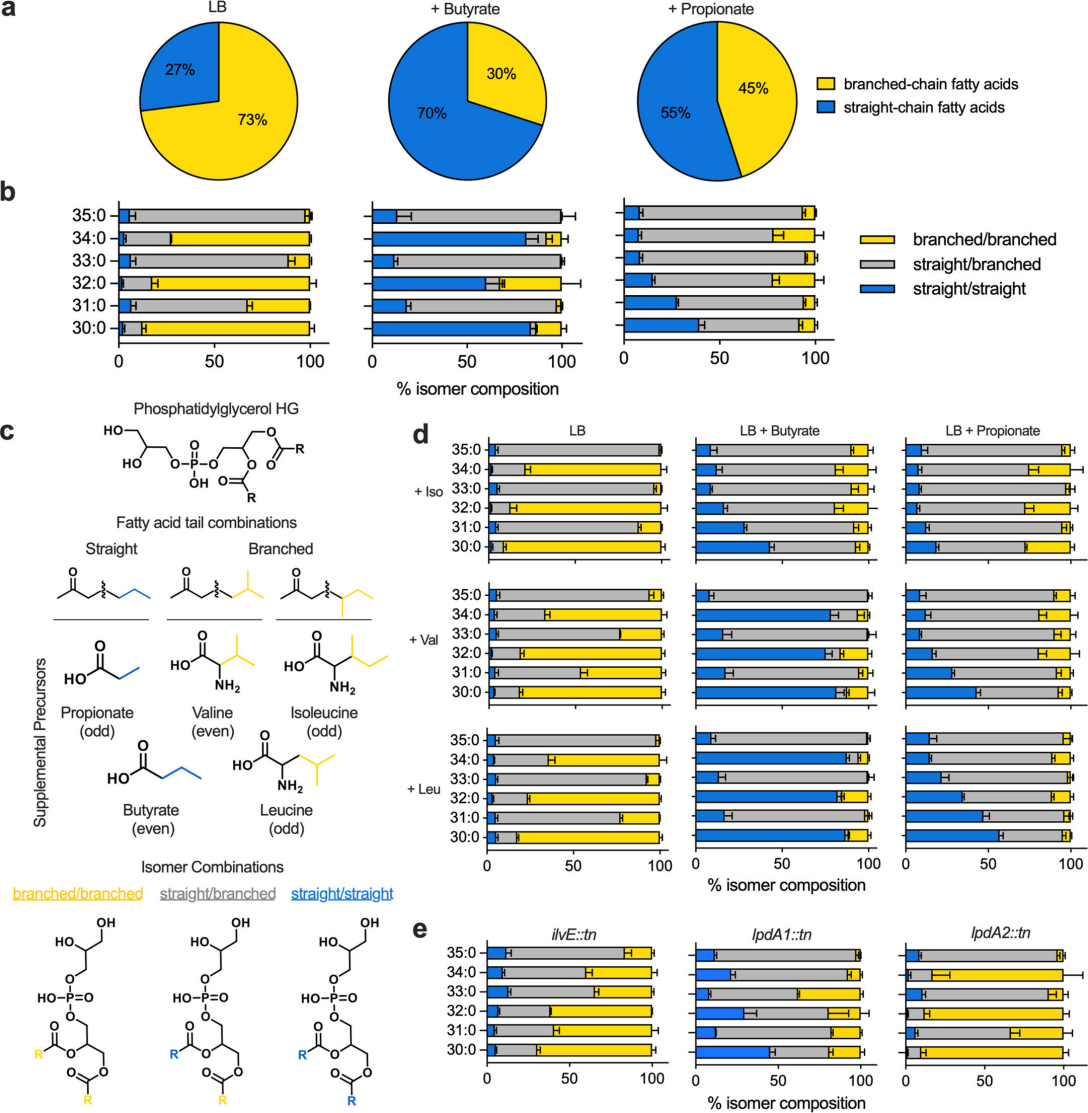
SCFAs alter *S. aureus* membrane lipid composition. (**a**) Ratio of branched-chain fatty acids to straight-chain fatty acids in *S. aureus* JE2 grown in LB with and without supplementation of sodium propionate or sodium butyrate. (**b**) Individual lipid isomer composition in LB or LB with propionate/butyrate supplementation. For each PG (30:0–35:0), three isomers (branched-branched, branched-straight, and straight-straight fatty acid combinations, shown in panel **c**, can be expected. Data are expressed as percentages of total PGs, as culture densities varied. (**d**) PG isomer percentage in LB, LB + propionate, and LB + butyrate supplemented with branched-chain amino acids isoleucine, valine, and leucine. (**e**) Isomer composition in JE2 transposon mutants (*ilvE::tn, lpdA1::tn, lpdA2::tn*) that were more sensitive to propionate and whose growth was not rescued by exogenous isoleucine.

Given the compromised membrane integrity in propionate and butyrate, we then performed targeted lipidomics to determine the membrane composition of JE2 when grown in LB with either SCFA, with or without BCAAs (Fig. 4). LB-grown *ilvE*::tn (sensitive to propionate, tolerant to butyrate), *lpdA1*::tn, and *lpdA2*::tn (sensitive to both SCFAs) were also evaluated. As predicted, growth of wild type in both SCFAs resulted in a markedly decreased BCFA:straight-chain FA ratio relative to LB alone (Fig. 4a; Fig. S5), though ratios of specific phosphatidylglycerol (PG)-acyl chain isomers (branched-branched, straight/branched, and straight-straight) varied between media (Fig. 4b and c). For example, cells grown in butyrate exhibited decreased PG head groups with two branched acyl chains (B/B) and increased abundances of two straight acyl chains (S/S) in the 34:0, 32:0, and 30:0 isomers (Fig. 4b). Growth in propionate resulted in similar reductions in B/B isomers but a large increase in S/B isomers and an increase in shorter chain S/S isomers. Consistent with growth curve data (Fig. 2), isoleucine supplementation of LB plus butyrate yielded increased S/B isomers, while leucine and valine supplementation led to increased S/S isomers, although chain lengths differed between media (Fig. 4d). Interestingly, *ilvE*::tn and *lpdA2*::tn mutants had branched/straight-chain FA ratios similar to wild type in LB alone, though the isomer composition for *ilvE*::tn was distinct (Fig. 4e). *ilvE*::tn had increased PG 34:0, 32:0, and 30:0 S/B isomers, while *lpdA1*::tn exhibited lower abundances of B/B isomers and increased S/B isomers relative to wild type and the other mutants. Isomer composition in *lpdA2*::tn was comparable to wild type, suggesting that its SCFA sensitivity is unrelated to BCFA metabolism. These data demonstrate that propionate and butyrate disrupt *S. aureus* lipid membrane homeostasis by altering BCFA metabolism, likely contributing to altered membrane integrity.

### SCFAs potentiate membrane-targeting antimicrobials

**Fig 5:**
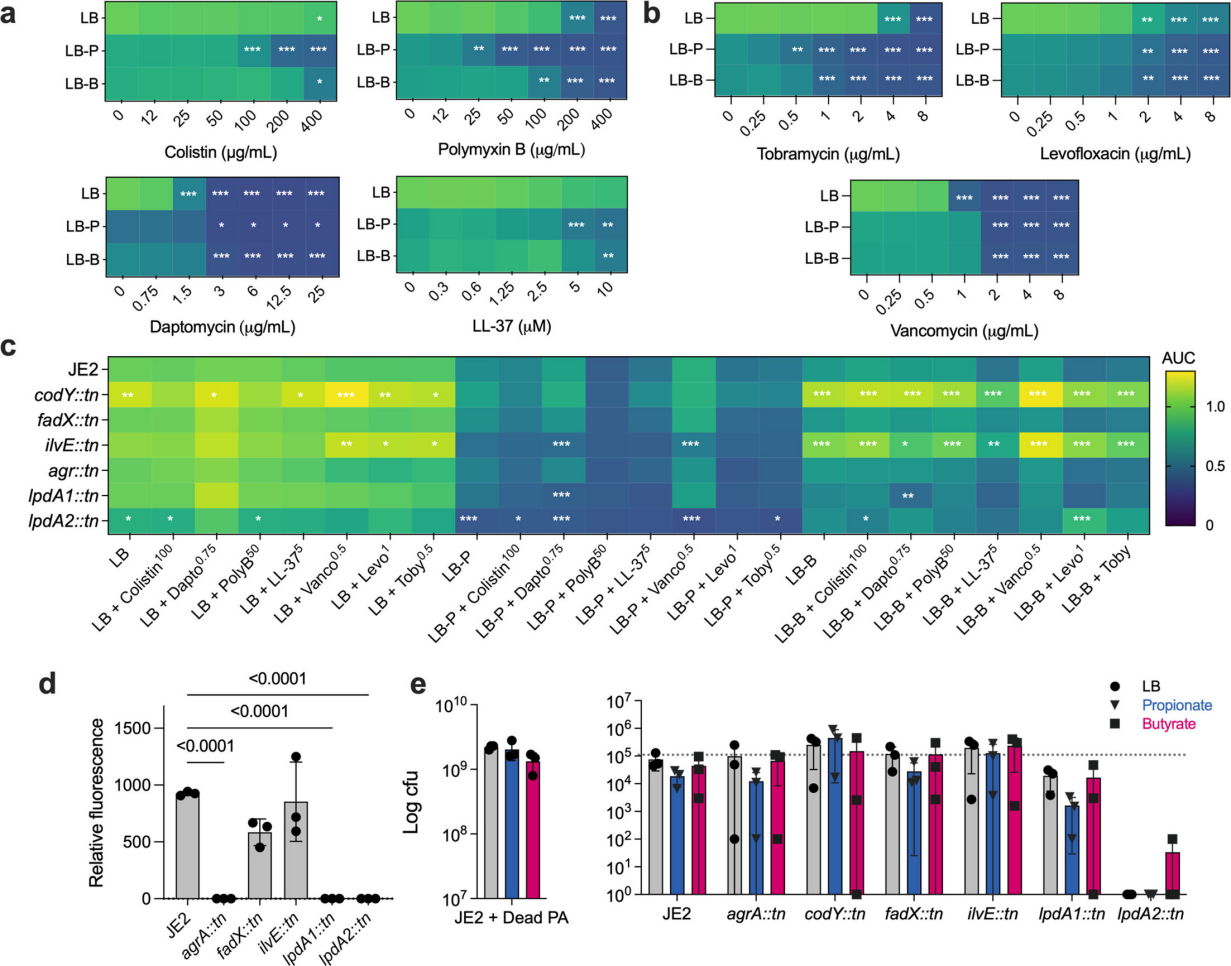
Altered BCFA metabolism by SCFAs sensitizes S*. aureus* to antimicrobials, impacts *agr* signaling, and reduces its fitness in competition with *P. aeruginosa*. (**a**) Heatmaps of normalized AUCs of JE2 grown in LB with or without propionate and butyrate, supplemented with escalating concentrations of colistin, polymyxin B, daptomycin, or the antimicrobial peptide LL-37. (**b**) Heatmaps of normalized AUCs of JE2 grown in LB with or without propionate and butyrate, supplemented with escalating concentrations of tobramycin, vancomycin, and levofloxacin. (**c**) Heatmap of normalized AUCs of JE2 and several transposon mutants in similar conditions as panel **a**, except only one dose of the antimicrobial was used. Data in panels **a, b, and c** were normalized to JE2 in LB and compared using a two-way ANOVA with Dunnett’s multiple comparison test (Data set S3). Additional growth curve data are shown in Fig. S6 and S7. (**d**) Relative fluorescence of *S. aureus* JE2 and various mutants carrying pAH1 after growth for 24 h in LB broth. Data were compared using an ordinary one-way ANOVA with Tukey’s multiple comparisons test. (**e**) *S. aureus* CFUs after 24 h of co-culture with *P. aeruginosa* PA14 on permeable membranes on LB agar plates with or without 10 mM sodium propionate or sodium butyrate. The left panel depicts JE2 co-cultured with isopropanol-killed PA14. Data for each mutant were compared using a one-way ANOVA with Dunnett’s multiple comparison test (Data set S3).

We next hypothesized that SCFA-induced lipid alterations would increase *S. aureus* sensitivity to membrane-targeting antimicrobials. To test this, JE2 was grown in LB supplemented with propionate and butyrate as before, but with escalating doses of colistin, polymyxin B, daptomycin, or the antimicrobial peptide LL-37. Each targets the bacterial membrane through distinct mechanisms and specificities. For example, polymyxin B and colistin perturb the membrane by displacing stabilizing cations (33) but are generally considered ineffective against *S. aureus* and other Gram-positive bacteria. Daptomycin targets Gram-positives by inserting its lipophilic tail into the membrane, driving depolarization via formation of ion-conducting pores (34). LL-37 acts on both Gram-positive and Gram-negative membranes via electrostatic interactions, forming disordered regions in the lipid bilayer and promoting leakage of cytoplasmic content (35). In LB alone, JE2 was resistant to colistin (up to 400 µg/mL) and LL-37 (up to 10 µM), but was inhibited by intermediate doses of daptomycin (>1.5 µg/mL) and polymyxin B (200 µg/mL) (Fig. 5a; Fig. S6). As predicted, JE2 exhibited greater sensitivity to each antimicrobial in the presence of SCFAs, suggesting a synergistic effect, with propionate being the more effective potentiator. Interestingly, we observed a similar potentiation with the aminoglycoside tobramycin, whose primary mode of action is translation inhibition but which can also destabilize membranes at high doses (Fig. 5b). However**,** synergy was not observed with levofloxacin or vancomycin, whose targets (DNA gyrase and peptidoglycan biosynthesis, respectively) are membrane-independent (36–38).

Transposon mutant sensitivity was consistent with previous experiments (Fig. 5c; Fig. S7). We selected a concentration for each antimicrobial that was permissive to growth of JE2 in LB but, when combined with one or both SCFAs, resulted in growth impairment. *ilvE*::tn, *lpdA1*::tn, and *lpdA2*::tn mutants exhibited worse growth than wild type in propionate plus antimicrobials. Consistent with their growth phenotypes in LB plus butyrate (Fig. 2c), *codY*::tn and *ilvE*::tn sensitivity to colistin, daptomycin, and polymyxin B was largely unaffected, though butyrate enhanced LL-37 activity relative to LB alone. These data suggest that despite differing spectra of activity and clinical use, SCFA-induced membrane alterations may increase membrane permeability to various antimicrobials, access to their respective targets, expose novel targets, and/or restrict antimicrobial defense mechanisms such as efflux.

### Altered BCFA metabolism impairs *agr* signaling

The *S. aureus agrBDCA* operon encodes the agr quorum-sensing system that regulates expression of numerous virulence factors (39). We previously showed that *agr* activity is compromised in the presence of SCFAs (11), leading us to hypothesize that loss of membrane homeostasis through reduced BCFAs also impairs agr signaling. To test this, we used a P3*_agr_-mCherry* reporter. While the wild type exhibited high relative fluorescence, the *lpdA1*::tn signal was undetectable, similar to the *agrA*::tn control (Fig. 5d). Despite having a branched:straight-chain FA ratio comparable to wild type (Fig. 4e), the *lpdA2*::tn mutant likewise exhibited no detectable fluorescence. In contrast, the propionate-sensitive *fadX*::tn mutant displayed intermediate P3*_agr_* activity compared to JE2 and *lpdA* mutants. Interestingly, *ilvE::*tn, which shows significant alterations to its membrane composition, retained fluorescence levels similar to wild type, though with greater variability across replicates. These data show that BCFA homeostasis is mechanistically linked to *S. aureus* quorum signaling. They also align with recent observations that *lpdA1*::tn displays reduced *agr* expression (21), further supporting the idea that SCFA-induced disruption of lipid metabolism impairs *S. aureus* virulence.

### *S. aureus*-*P. aeruginosa* competition is altered by BCFA metabolism

Pathogen colonization of the airways occurs in a complex milieu of microbiota and the metabolites they exchange. *S. aureus* and *P. aeruginosa* coinfection of the airways is well documented, as is competition between them, with both organisms possessing mechanisms that influence each other’s fitness (40–42). Here, we tested the hypothesis that SCFAs tip the competitive balance in favor of *P. aeruginosa* by compromising *S. aureus* membrane integrity.

To test this, we co-cultured *P. aeruginosa* PA14 with *S. aureus* JE2 or transposon mutants on LB agar with or without 25 mM propionate or butyrate (elevated concentrations impaired *P. aeruginosa* growth) (Fig. 5e). When compared to co-culture with isopropanol-killed PA14, ~4 logs fewer *S. aureus* CFUs were recovered when live bacteria were used, confirming robust PA14-driven effects on *S. aureus* viability. Co-culture on propionate resulted in a fourfold reduction in viable *S. aureus* compared to LB, though it was not statistically significant; CFUs were also lower on butyrate but variable between replicates.

Mutant analysis revealed differential sensitivities. *codY*::tn survived co-culture with PA14 on LB and LB + butyrate similarly to JE2 in both conditions, but CFUs were higher on propionate. *fadX*::tn showed no differences in viability under these conditions relative to wild type, while *ilvE*::tn was also unaffected by either SCFA. *lpdA1*::tn was more sensitive to PA14-mediated killing than JE2 in both SCFA media compared to LB alone, though neither condition was statistically significant. Finally, *lpdA2*::tn was markedly less fit in co-culture with PA14, being at or below the level of detection (10^2^ CFU) in all three media. These data indicate that SCFAs influence *S. aureus* competitive fitness and that BCFA metabolism contributes to its ability to resist killing by *P. aeruginosa*.

### SCFA-mediated phenotypes are conserved in CF and CRS clinical isolates of *S. aureus*

**Fig 6:**
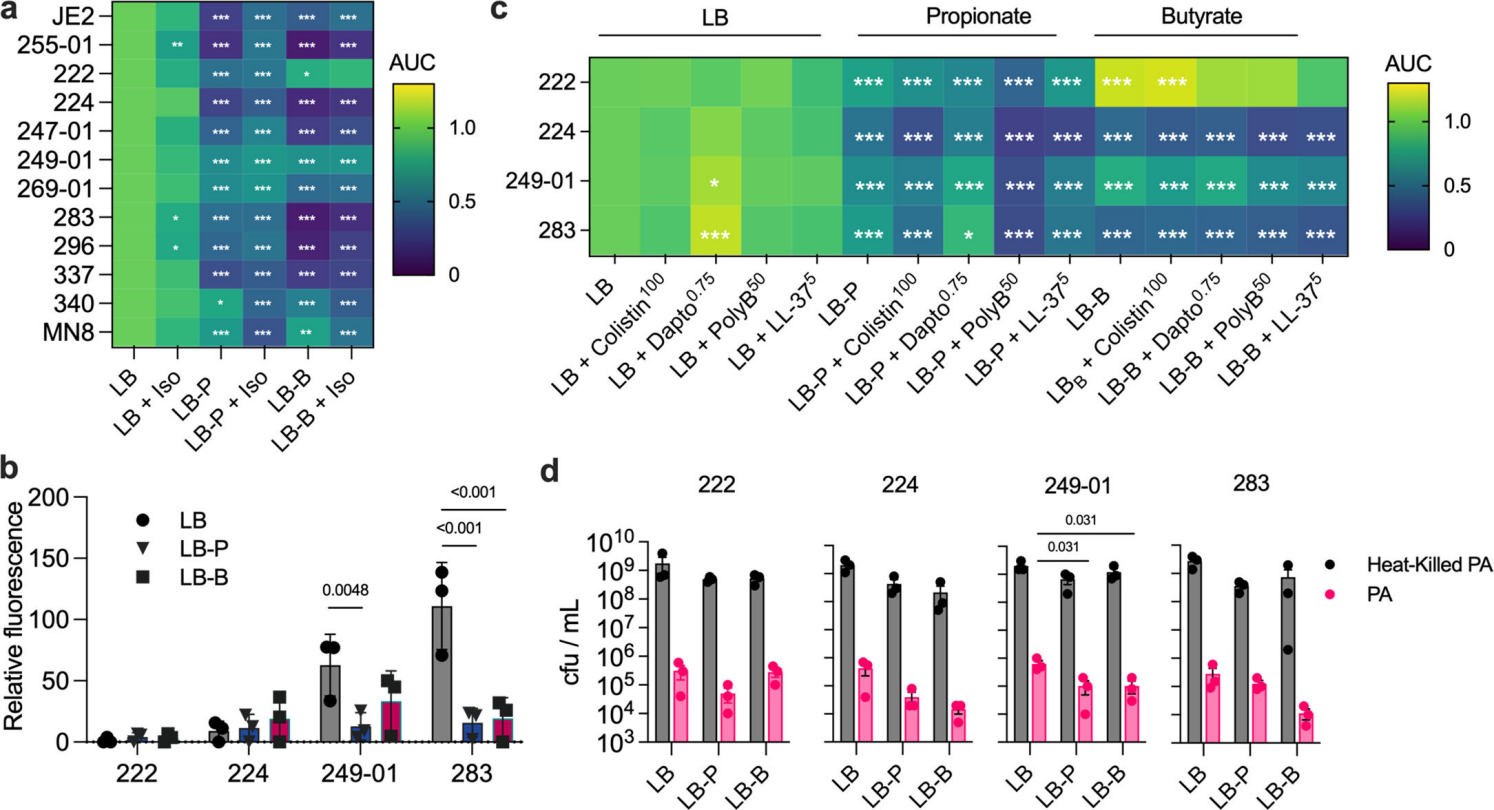
SCFA-mediated lipid remodeling phenotypes are conserved across *S. aureus* clinical isolates. (**a**) Ten clinical isolates of *S. aureus* from patients with CF or CRS and one from a patient with toxic shock syndrome (MN8) were grown in LB with propionate or butyrate, with and without isoleucine supplementation. Data represent the mean of *n* = 3 biological replicates of each isolate normalized to the first column (LB alone) using a two-way ANOVA with Dunnett’s multiple comparison test (*, *P* < 0.05; **, *P* < 0.01; ***, *P* < 0.001). (**b**) Fluorescence of clinical isolates carrying the P3*_agr_-mCherry* reporter plasmid pAH1 when grown in LB with or without SCFA supplementation (*n* = 3 biological replicates). Data for each isolate were compared using a two-way ANOVA with Tukey’s multiple comparisons test. (**c**) Antimicrobial susceptibility of clinical isolates with SCFA supplementation (*n* = 3 biological replicates). Data in each row were normalized to the first column (untreated clinical isolate in LB) and compared using a two-way ANOVA with Dunnett’s multiple comparison test (*, *P* < 0.05; **, *P* < 0.01; ***, *P* < 0.001). (**d**) Fitness in competition with *P. aeruginosa* PA14 with and without the presence of SCFAs. Statistical comparisons in each panel were too numerous to depict graphically and are shown in Data set S3.

JE2 is a derivative of USA300 LAC that was originally isolated from a skin abscess. Therefore, we cultured a panel of *S. aureus* isolates from airway mucus and performed assays under identical growth conditions (Fig. 6). A toxic shock syndrome isolate (MN8) was also included as a second outgroup derived from a non-airway infection. We observed varied SCFA sensitivities: some strains resembled JE2 (224 and 247-01), while others were more (222, 249-01, 269-01, 337, 340, MN8) or less (255-01, 283, 296) tolerant (Fig. 6a; Fig. S8). Notably, isoleucine supplementation generally improved *S. aureus* growth in LB with either SCFA, further supporting that propionate and butyrate disrupt its BCFA metabolism.

Focusing on strains with growth phenotypes distinct from JE2 (222, 224, 249-01, 283), we examined AgrBDCA activity using the P3*_agr_-mCherry* reporter. Neither 222 nor 224 fluoresced (Fig. 6b), indicating nonfunctional Agr systems, a known characteristic of some clinical isolates (43, 44). In contrast, 249-01 and 283 exhibited *agr*-mediated fluorescence, though at lower levels than JE2, which SCFA supplementation suppressed.

SCFA effects on antimicrobial sensitivity in SCFAs were variable. Daptomycin had no impact on 222 in butyrate, but slightly increased growth of 224, 249-01, and 283 in propionate (Fig. 6c; Data set S3). Strain 222 exhibited enhanced growth in butyrate supplemented with colistin relative to butyrate alone, although daptomycin, polymyxin B, and LL-37 reduced its growth. Additionally, SCFAs impaired the competitive fitness of *S. aureus* against *P. aeruginosa;* viability of 222, 224, and 249-01 was lower from co-cultures on propionate relative to LB, as were those from 224, 249-01, and 283 on butyrate (Fig. 6d). Collectively, these data support the hypothesis that SCFAs broadly impact *S. aureus* physiology by disruption of membrane homeostasis.

## DISCUSSION   

The emerging picture of airway colonization by strict and facultative anaerobes raises questions about their contributions to disease and interactions with canonical respiratory pathogens like *S. aureus*. A key class of metabolites produced by these anaerobes is SCFAs, which accumulate as byproducts of carbohydrate and amino acid fermentation (5, 10). Building on previous findings (11, 13, 45), here, we used genetic and multi-omic approaches to show that propionate and butyrate impair *S. aureus* growth, alter membrane lipid composition and polarization, suppress agr quorum sensing, increase antimicrobial sensitivity, and reduce its fitness in competition with *P. aeruginosa*. Many of these effects were mitigated by adding BCAAs in excess to the growth medium. Considered altogether, our data suggest that the observed phenotypes are due to alterations of the abundances of specific iso and anteiso BCFAs in the membrane, likely resulting in inhibition of BCFA-responsive membrane protein function (21).

BCFAs comprise the majority of lipid species in the *S. aureus* membrane when grown in common bacteriological media such as LB. The BCFA-to-SCFA ratio is crucial for maintaining membrane homeostasis and responding to environmental perturbation (16, 17, 19, 20). *S. aureus* BCFA mutants can bypass BCFA auxotrophy *in vitro* due to the presence of short BCFAs in LB and *in vivo* by acquiring host-derived unsaturated fatty acids through Geh lipase activity (46). While host lipid acquisition could potentially help *S. aureus* overcome growth inhibition by propionate and butyrate, the role of Geh in *S. aureus* pathogenesis in chronic airway disease remains unclear. Notably, *geh* was not induced in endogenous *S. aureus* added to CF sputum compared to *in vitro* conditions, nor was it responsive to SCFAs *in vitro* (Fig. 1) (45, 47). Beyond altering membrane lipids, propionate and butyrate may also impact *S. aureus* physiology by disrupting BCFA-dependent membrane protein functions, as observed with the SaeRS two-component system (21). This raises the intriguing question of how *S. aureus* senses membrane composition and adjusts its metabolism to maintain homeostasis across a variety of host environments.

The impact of SCFAs on membrane lipid composition underscores the importance of BCAA availability in the mammalian host as a key source of essential BCFA precursors. Kaiser et al. demonstrated that *S. aureus* relies on BCAA uptake via BcaP and BrnQ1 for persistence in a murine nasal colonization model (48). However, this study was conducted in mice pretreated to deplete endogenous microbiota. In a competitive microbial environment with limited nutrients, SCFA-producing anaerobes in close proximity to *S. aureus* may reduce its fitness by slowing growth and suppressing quorum-regulated virulence factors, a process likely exacerbated by BCAA availability. Additionally, short BCFA precursors are also abundant in the human gut and may serve as a reservoir of precursors to full-length BCFAs, potentially helping *S. aureus* colonize the GI tract by overcoming growth restrictions imposed by high SCFA concentrations produced by endogenous microbiota (49).

SCFAs may also modulate the host response. In a murine model, instillation of micro-to-millimolar concentrations of propionate to the lungs prior to challenge with luminescent *S. aureus* led to increased luminescence in treated mice relative to untreated mice after 6 h. This suggested enhanced *S. aureus* growth due to a blunted inflammatory response (50). Conversely, *in vitro* studies show that CF epithelial cells treated with SCFAs secrete more IL-8 than non-CF cells (10). In addition to SCFAs produced locally in the airways by anaerobes, systemically circulating SCFAs produced by gut microbiota may also influence pathogen colonization and the host immune response. Indeed, propionate and butyrate protected mice against *S. aureus* in an experimental mastitis model by strengthening the blood-milk barrier (51).

The reductionist nature of our *in vitro* approach necessarily omits additional features of the airways, including spatial structure, host contributions, and the metabolic complexity of polymicrobial communities. Although we observed SCFA-mediated effects on *S. aureus*, the extent to which these findings translate to the *in vivo* environment will require further study. In these environments, SCFA production likely depends on the abundance and spatial proximity of anaerobes in mucus-occluded, hypoxic regions, where oxygen gradients favor mixed-acid fermentation. Additional factors, including mucus viscosity, host inflammatory responses, and metabolic cross-feeding, likely modulate local SCFA levels and their impact on traditional pathogens. Our use of supraphysiological SCFA concentrations in rich media was intended to identify membrane-associated responses that may occur under more complex conditions. While not a direct proxy for CF or CRS mucus, these findings motivate future studies using more complex and physiologically relevant models, such as airway epithelial cultures or animal models that might better capture the spatial and metabolic constraints that govern SCFA availability. Given the biocompatibility of SCFAs and their ability to potentiate antimicrobial activity, we propose that these and other commensal-derived metabolites warrant further investigation as modulators of *S. aureus* behavior in polymicrobial host environments.

## MATERIALS AND METHODS

### Bacterial strains

*S. aureus* JE2 and mutants from the Nebraska Transposon Mutant Library (52) were cultured in LB at 37°C with shaking (220 rpm). Erythromycin (4 µg/mL) and chloramphenicol (10 µg/mL) were added for transposon mutants or strains carrying pAH1, respectively. Strains are listed in Table S1. Clinical isolates were recovered from mucus specimens on mannitol salt agar (MSA). Transposon mutants were confirmed by whole-genome sequencing (10.6084/m9.figshare.29955194).

### Clinical specimens

Sinus secretions collected during functional endoscopic sinus surgery and expectorated CF sputum were obtained from patients at the University of Minnesota.

### Growth assays

Overnight cultures of JE2 and transposon mutants were diluted 1:100 in PBS, from which 5 µL was added to 195 µL of media in a 96-well plate. Media were LB, LB + 100 mM of either sodium propionate or sodium butyrate, LB supplemented with 1 mg/mL of one BCAA (isoleucine, leucine, or valine), or LB supplemented with either SCFA and one of the three BCAAs, unless otherwise indicated. Additional assays were performed in LB + SCFA (50 mM) with antibiotics (colistin sulfate, polymyxin B, daptomycin, vancomycin, tobramycin, levofloxacin) or cathelicidin (LL-37). For daptomycin, 50 µg/mL of calcium chloride was added to both LB and LB plus daptomycin. The 96-well plates were incubated in a BioTek Synergy H1 microplate reader at 37°C with hourly readings at OD_600_. Area under the curve was calculated using GraphPad Prism.

### Membrane integrity

Overnight cultures were diluted 1:100 into LB or 1:50 in LB + SCFAs. The 1:50 dilution was used to account for SCFA-induced growth impediment. Cultures were incubated at 37°C with shaking at 220 rpm. After 4 h, cultures were adjusted to OD_600_ = 0.3 using the same media, and 195 µL was added to black, clear-bottom, 96-well plates blocked with 5% BSA (53, 54). Cultures treated with carbonyl cyanide 3-chlorophenylhydrazone (15 µM final concentration) were used as a control. Five microliters of a 40 µM stock solution of DiSC_3_(5) in 0.1% DMSO was added to each well (final concentration 1 µM) and quantified using excitation/emission wavelengths of 651 nm and 675 nm, respectively.

For LIVE/DEAD (Invitrogen L7012) staining, overnight cultures were diluted as described above and incubated at 37°C with shaking at 220 rpm. After 4 h, cultures were centrifuged at 8,000 × *g* for 10 min. Pellets were washed three times with 0.85% NaCl and incubated at room temperature for 1 h. Cells were stained with SYTO9/propidium iodide mix (1.5 µL of each per mL of cells in 0.85% NaCl) and incubated in the dark for 15 min. One hundred microliters was added to a 96-well plate in triplicate. Fluorescence was quantified using an excitation wavelength of 485 nm and emission wavelengths of 530 nm (green) and 630 nm (red).

### Transmission electron microscopy

JE2 grown in LB, LB + 50 mM sodium propionate, or LB + 50 mM sodium butyrate was diluted 1:100 in fresh media, grown for an additional 3 h, and centrifuged for 10 min at 8,000 × *g*. Pellets were washed three times with 50 mM HEPES, enrobed in 2% Noble agar, cut into 1–2mm blocks, and fixed with 2% glutaraldehyde in HEPES for 2 h. Blocks were then fixed *en bloc* using 2% (wt/vol) osmium tetroxide in HEPES for 2 h and stained with 1% (wt/vol) uranyl acetate for 1 h. Samples were serially dehydrated in 25%, 50%, 75%, and 95% ethanol for 15 min each, followed by three incubations in 100% ethanol. Blocks were suspended in a 50:50 ethanol:LR White resin for 2 h followed by 100% LR White for 2 h. Blocks were embedded in gelatin capsules containing LR White and polymerized at 60°C for ~4 h. Specimens were thin-sectioned on a Reichert-Jung Ultracut E microtome, mounted on carbon-coated 200 mesh grids, and imaged on a Hitachi HT7800 120 kV TEM. Peptidoglycan thickness was quantified as described previously (32).

### Nanostring

Overnight LB cultures of *S. aureus* were diluted 1:500 and grown to an OD_600_ of ~0.2–0.3. Cells were pelleted by centrifugation at 14,000 rpm for 1 min, resuspended in 50 µL of LB + 20 µg/mL lysostaphin (Sigma-Aldrich), and incubated at 37°C for 15 min. Lysates were dissolved in 1 mL of TRIzol Reagent for 5 min, followed by the addition of 200 µL chloroform. Samples were agitated for 15 s, incubated for 5 min, and then centrifuged at 12,000 rpm for 15 min at 4°C. The aqueous phase was mixed with an equal volume of 95% ethanol and vortexed for 5 s. The mixture was then subjected to on-column DNase I treatment using the Zymo RNA Clean & Concentrator kit. RNA was submitted to the UMN Genomics Center where it was hybridized to a custom Nanostring codeset (11). Expression data were analyzed using nSolver software. Z-scores were calculated as (sample value – group mean) / group standard deviation. Transcripts with a Benjamini-Hochberg-adjusted *P*-value <0.05 were considered significant.

### Proteomics

JE2 was grown to an OD_600_ of ~0.3 and labeled for tandem mass tag proteomics. Cells were treated with lysis buffer (7 M urea, 2 M thiourea, 0.8 M triethylammonium bicarbonate, 20% acetonitrile, 4 mM tris(2-carboxyethyl)phosphine), followed by probe sonication (7 s at 30% amplitude on ice) and high-pressure barocycling (35 psi for 20 s, 0 psi for 10 s, 60 cycles). Lysates were treated with 200 mM 2-chloroacetamide for 15 min. Desalting was achieved using MCX stage tips. Labeled peptide fractions were run on an Orbitrap Eclipse mass spectrometer with high-energy collision-induced dissociation for ion mobility spectrometry. Data were analyzed via Proteome Discoverer using the UniProt *Staphylococcus aureus* pan-proteome database (UP000008816) merged with contaminant sequences (thegpm.org/crap/).

### Lipidomics

Membrane lipids were extracted using a modified Bligh and Dyer method (55) and evaluated by mass spectrometry, as previously described (32, 56).

### *agr* reporter activity

P3*_agr_-mCherry* reporter activity was assayed as described previously. Briefly, JE2, transposon mutants, and clinical isolates were electroporated with pAH1 and plated on LB with 10 µg/mL chloramphenicol. Isolated colonies were grown at 37°C for 24 h with shaking at 220 rpm. Two hundred microliters from each culture was added to a black-walled, flat-bottom 96-well plate. Fluorescence was measured using excitation and emission wavelengths of 580 nm and 635 nm, respectively. Fluorescence was normalized by OD_600_, and technical replicates were averaged to yield the relative fluorescence for each biological replicate (*n* = 3).

### Competition assays

Competition assays were performed using established protocols (57). Overnight cultures of *S. aureus* (JE2, transposon mutants, *S*. clinical isolates) and *P. aeruginosa* PA14 were sub-cultured 1:5 in fresh LB and grown for another 2 h. OD_600_ was determined and cultures were centrifuged at 5,000 rpm for 5 min. Pellets were suspended in 1 mL of PBS, then diluted in PBS to OD_600_ = 0.01 (*S. aureus*) and OD_600_ = 0.02 (*P. aeruginosa*), resulting in ~10^7^ CFU/mL of each bacterium. In parallel, one culture of PA14 was centrifuged and suspended in 1 mL of isopropanol for 30 min and washed three times in PBS to create a dead control. For the competition assay, 50 µL each of PA14 and *S. aureus* was combined and vortexed for 5 s. Five microliters was spotted onto a Nucleopore Track-Etch membrane (0.2 µm pore size, 13 mm diameter) on LB agar. Plates were incubated agar-side up at 37°C for 24 h. Membranes were suspended in 1 mL PBS, vortexed for 5–10 s, and allowed to sit on the benchtop for ~10 min, followed by another 5–10 s of vortexing and manual disruption via pipetting. Samples were serially diluted in PBS and plated onto MSA for enumeration.

## Data Availability

Nanostring data and related files are available at http://github.com/Hunter-Lab-UMN/Fletcher_et_al_2025 and the NCBI Gene Expression Omnibus (GSE309823). Proteomics raw data are deposited to the Mass Spectrometry Interactive Virtual Environment (MassIVE) database (accession MSV00099871) and ProteomeXchange (PXD070658).

## SUPPLEMENTAL MATERIAL

Data set S1 mbio.01392-25-s0001.xlsx Nanostring gene expression data.

Data set S2 mbio.01392-25-s0002.xlsx Proteomics data.

Data set S3 mbio.01392-25-s0003.xlsx Comprehensive statistical analyses.

Supplemental Material mbio.01392-25-s0004.pdf Figures S1 to S10; Table S1.

ASM does not own the copyrights to Supplemental Material that may be linked to, or accessed through, an article. The authors have granted ASM a non-exclusive, world-wide license to publish the Supplemental Material files. Please contact the corresponding author directly for reuse.
