# Supplementary material for: Commensal-derived short-chain fatty acids disrupt lipid membrane homeostasis in Staphylococcus aureus
Source: mBio. 2025 Nov 28;17(1):e01392-25. doi: 10.1128/mbio.01392-25 (PMC12802256; doi:10.1128/mbio.01392-25)
Supplement: Supplemental Material — Figures S1 to S10; Table S1. [file mbio.01392-25-s0004.pdf]

Supplemental Material for:

Commensal-derived short-chain fatty acids disrupt lipid membrane homeostasis in *Staphylococcus aureus*

Joshua R. Fletcher, Lisa A. Hansen, Julia R. Hoyser, Allison E. Hanna, Richard Martinez, Christian Freeman, Niall T. Thorns, Mitchell R. Penningroth, Alex R. Villareal, Grace A. Vogt, Matthew Tyler, Kelly M. Hines, Ryan C. Hunter

Ryan C. Hunter
Email: rhunter2@buffalo.edu

This PDF file includes:

Figures S1 to S10
Table S1
Legends for Datasets S1 to S3
SI References

Other supporting materials for this manuscript include the following:

Datasets S1, S2, S3

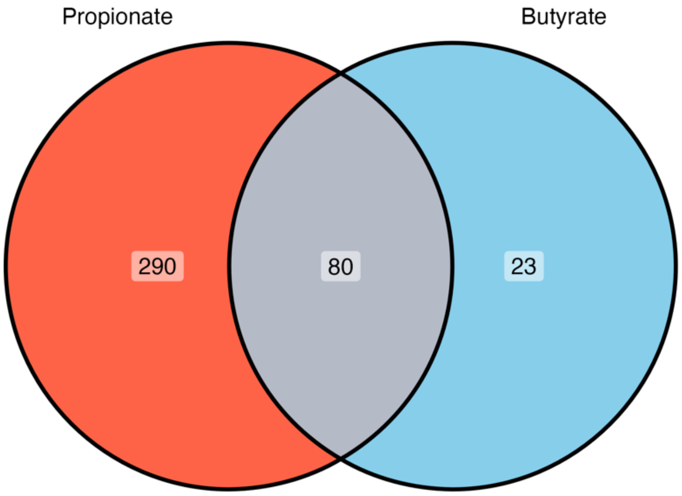

Figure S1. Venn diagram depicting differentially expressed *S. aureus* proteins during growth in LB+propionate and LB+butyrate relative to LB alone. 80 proteins were differentially expressed in the presence of either SCFA.

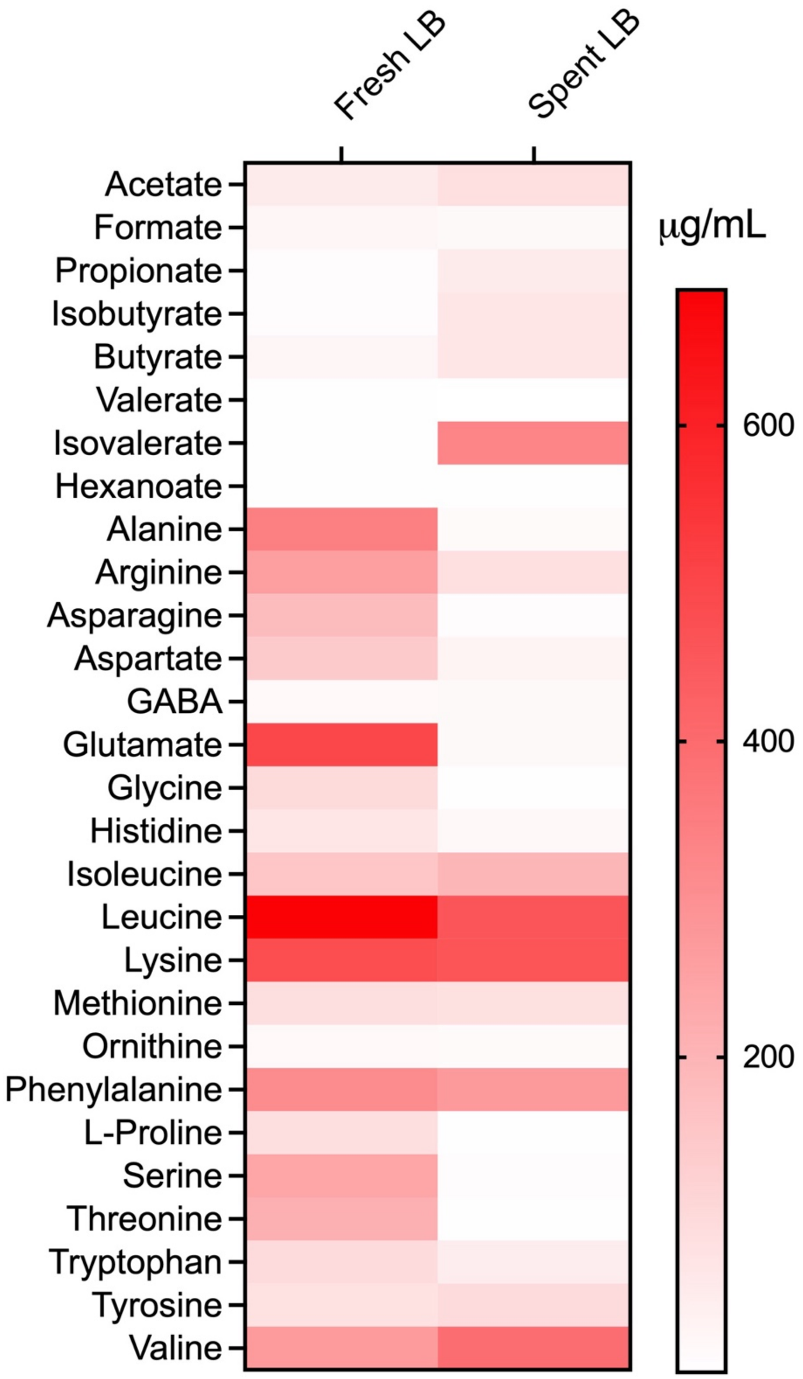

Figure S2. LB medium, before (fresh) and after *S. aureus* JE2 growth (spent), was filter-sterilized and sent to Creative Proteomics for targeted LC-MS and GC-MS metabolomic analysis of amino acid and short-chain fatty acid content, respectively. Data represent the mean of three replicate medium preparations and spent *S. aureus* supernatants. Standard curves were used to calculate the concentration of SCFAs in the samples from the peak area detected.

Table S1. Strains used throughout this study.

Staphylococcus aureus

Strain	Locus tag	NTML ID[1]	BEI ID	Source[2]
JE2			NR-46543	1
agrA::tn	SAUSA300_1992	NE1532	NR-48074	1
bcaP::tn	SAUSA300_2538	NE206	NR-46749	1
brnQ1::tn	SAUSA300_0188	NE945	NR-47488	1
brnQ2::tn	SAUSA300_0306	NE605	NR-47148	1
brnQ3::tn	SAUSA300_1300	NE44	NR-46587	1
codY::tn	SAUSA300_1148	NE1555	NR-48097	1
crtM::tn	SAUSA300_2499	NE1444	NR-47986	1
fadX::tn	SAUSA300_0229	NE263	NR-46806	1
fakA::tn	SAUSA300_1119	NE229	NR-46772	1
fakB1::tn	SAUSA300_0733	NE1540	NR-48082	1
fakB2::tn	SAUSA300_1318	NE403	NR-46946	1
ilvD::tn	SAUSA300_2006	NE718	NR-47261	1
ilvE::tn	SAUSA300_0539	NE292	NR-46835	1
leuB::tn	SAUSA300_2011	NE76	NR-46619	1
lpdA1::tn	SAUSA300_1467	NE1896	NR-48438	1
lpdA2::tn	SAUSA300_0996	NE1610	NR-48152	1
pdhB::tn	SAUSA300_0994	NE1758	NR-48300	1

Strain	Description	Source[2]
MN8	Methicillin sensitive isolate from a patient with toxic shock syndrome	3
222	Clinical isolate from patient with chronic sinusitis	4
224	Clinical isolate from patient with chronic sinusitis	This study
247-01	Clinical isolate from patient with chronic sinusitis	This study
249-01	Clinical isolate from patient with chronic sinusitis	This study
255-01	Clinical isolate from patient with chronic sinusitis	This study
269-01	Clinical isolate from patient with chronic sinusitis	This study
283	Clinical isolate from patient with chronic sinusitis and cystic fibrosis	This study
296	Clinical isolate from patient with chronic sinusitis	This study
337	Clinical isolate from patient with chronic sinusitis	This study
340	Clinical isolate from patient with chronic sinusitis	This study

Pseudomonas aeruginosa

Strain	Description	Source
PA14	UCBPP-PA14, burn wound isolate	5

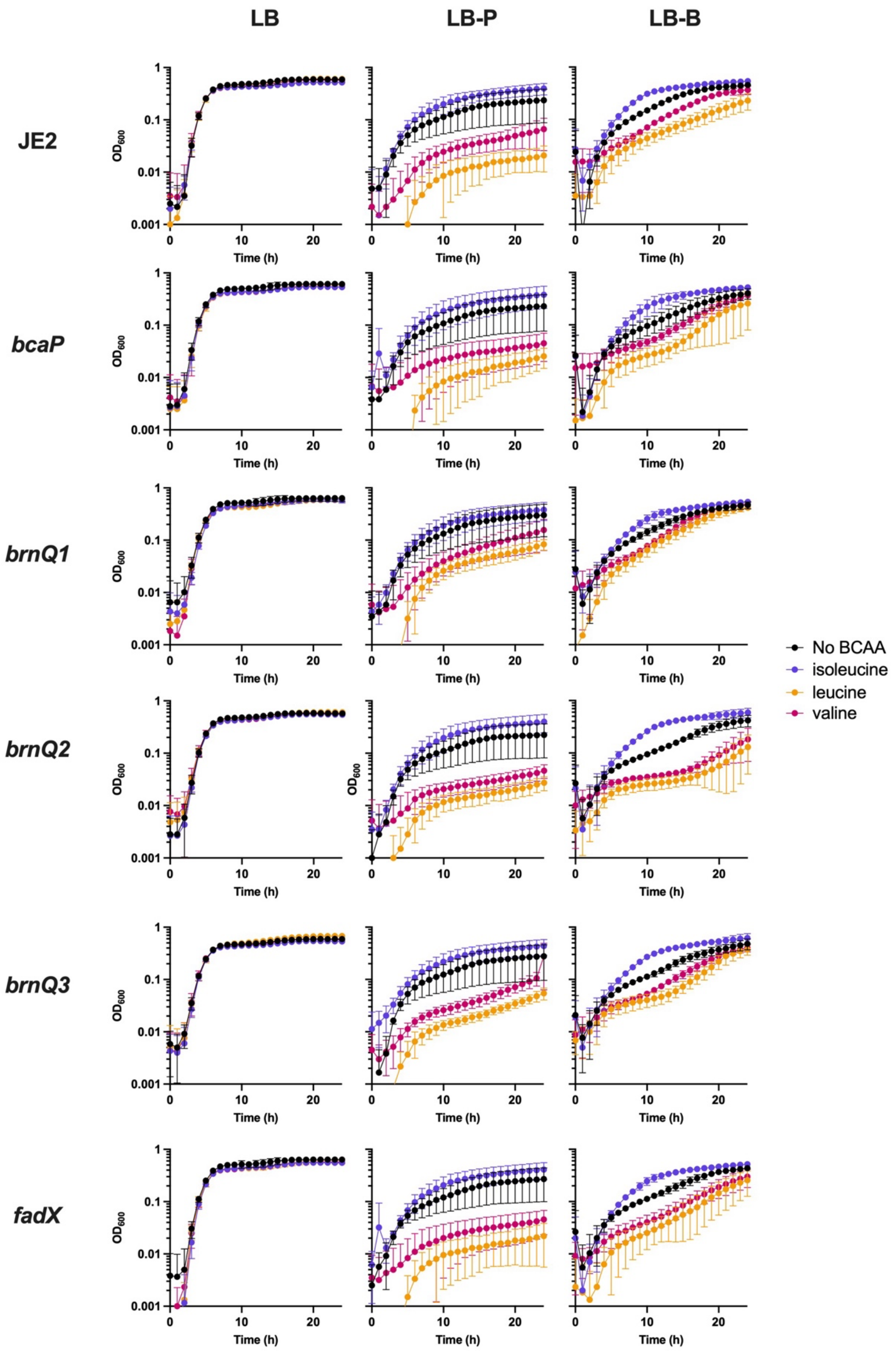
LB
LB-P
LB-B
JE2
bcaP
brnQ1
brnQ2
brnQ3
fadX
OD600
Time (h)
No BCAA
isoleucine
leucine
valine

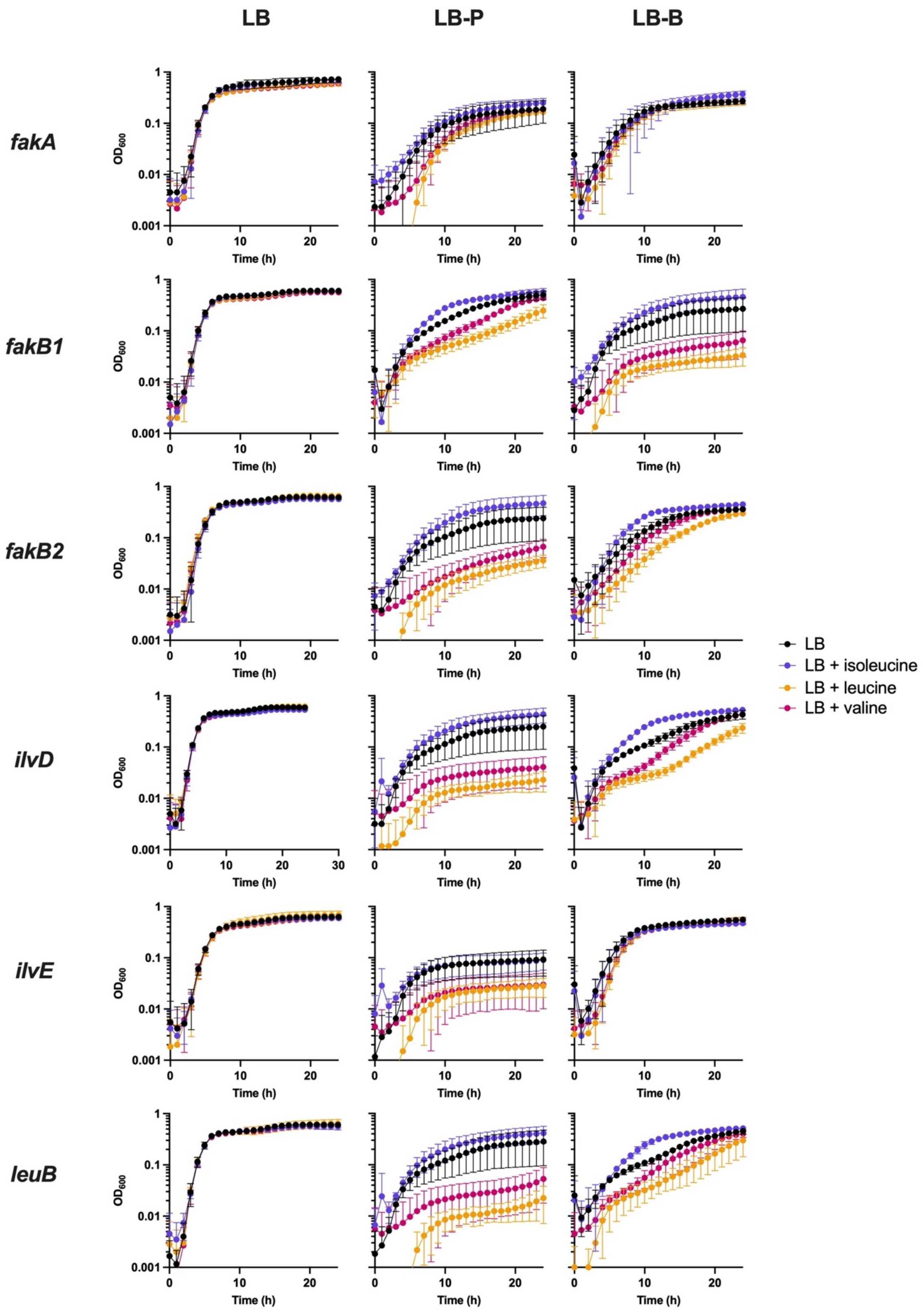
LB
LB-P
LB-B
fakA
fakB1
fakB2
ilvD
ilvE
leuB
OD600
Time (h)
LB
LB + isoleucine
LB + leucine
LB + valine

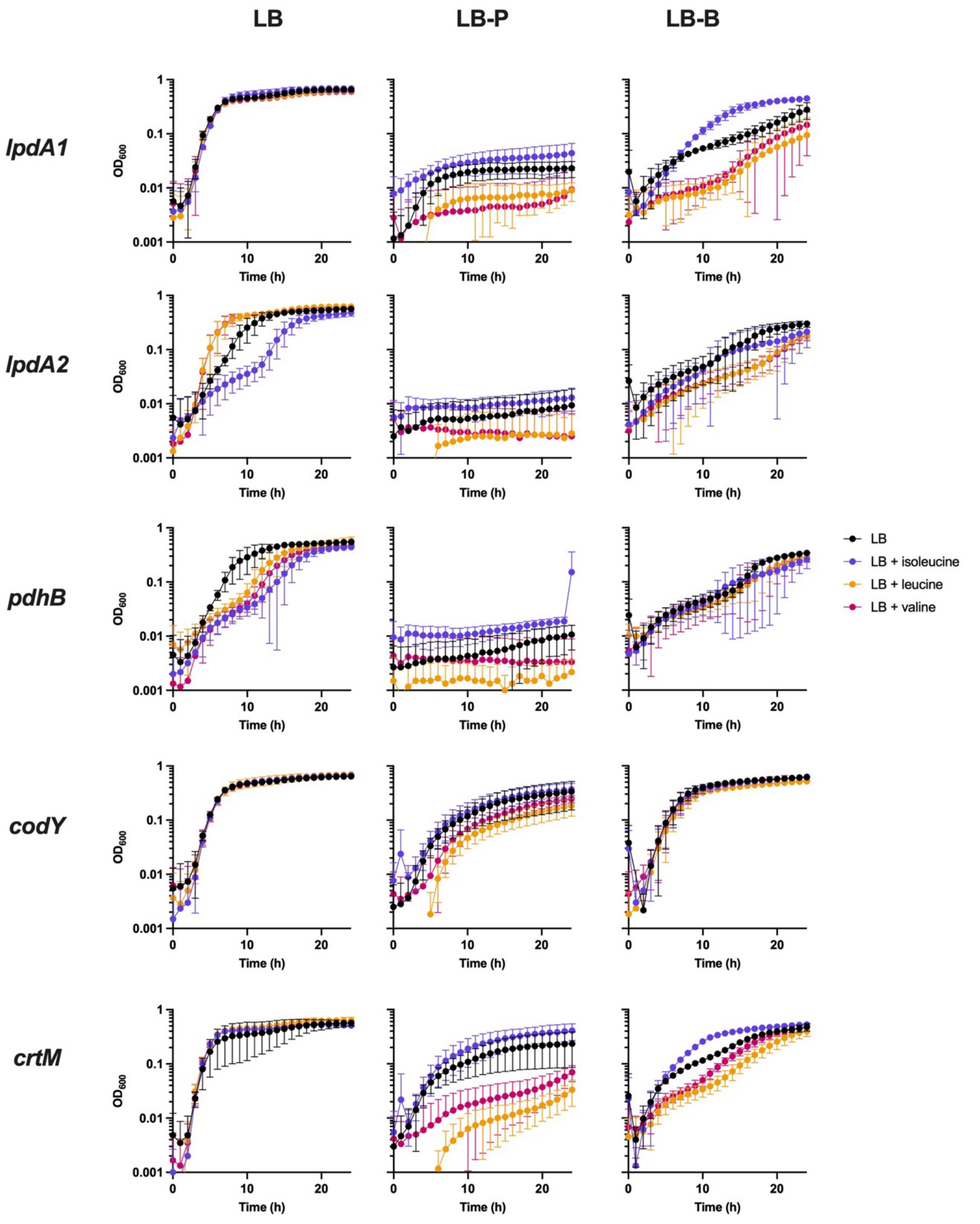

Figure S3. Raw growth curve data for normalized AUC heatmap shown in Figure 2. Data represent growth of *S. aureus* JE2 and transposon mutants in genes associated with BCAA uptake/metabolism or fatty acid metabolism. Each strain was grown LB +/- propionate or butyrate, +/- one branched-chain amino acid. n=6 growth curves per strain, per condition.

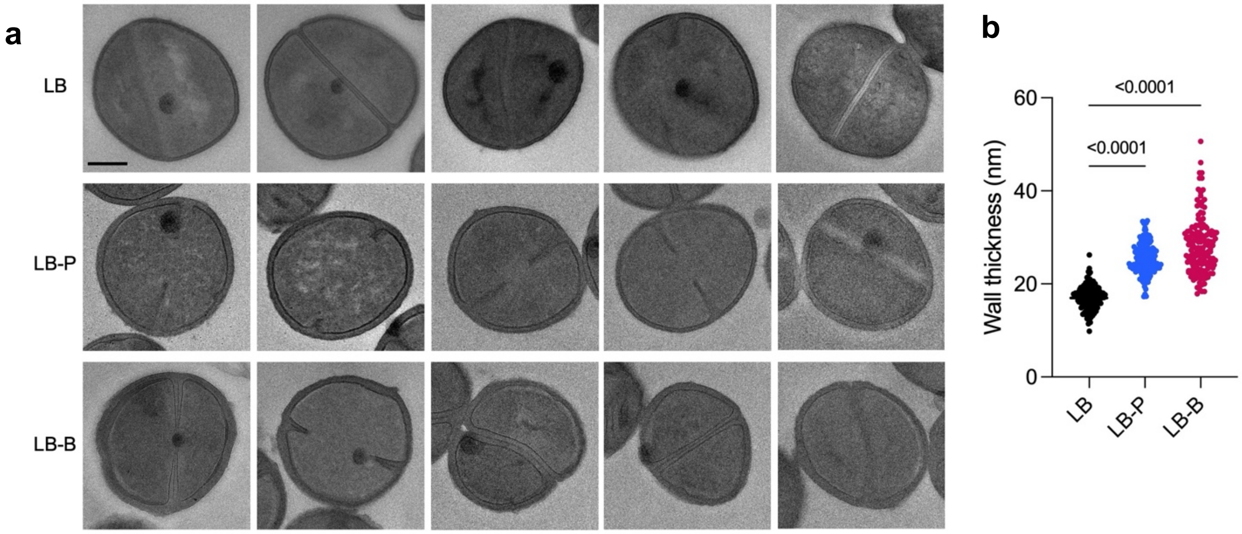

Figure S4. Propionate and butyrate alter cell wall but not cytoplasmic membrane ultrastructure. (a) Representative electron micrographs of *S. aureus* JE2 grown in LB (top), LB + propionate (middle), and LB + butyrate (bottom). Bar= 200nm. Growth in SCFA induced increased cell wall thickness consistent with prior work, however no obvious differences in membrane architecture were observed relative to LB alone. **(b)** Cell wall thickness for *S. aureus* JE2. Images are representative of n=3 biological replicate cultures of each condition. Cell wall data were taken from 5 cells per condition, 30 measurements per cell, and represent 150 individual measurements for each growth condition as previously described (6). Data were compared using a one-way ANOVA with Dunnett's multiple comparisons test.

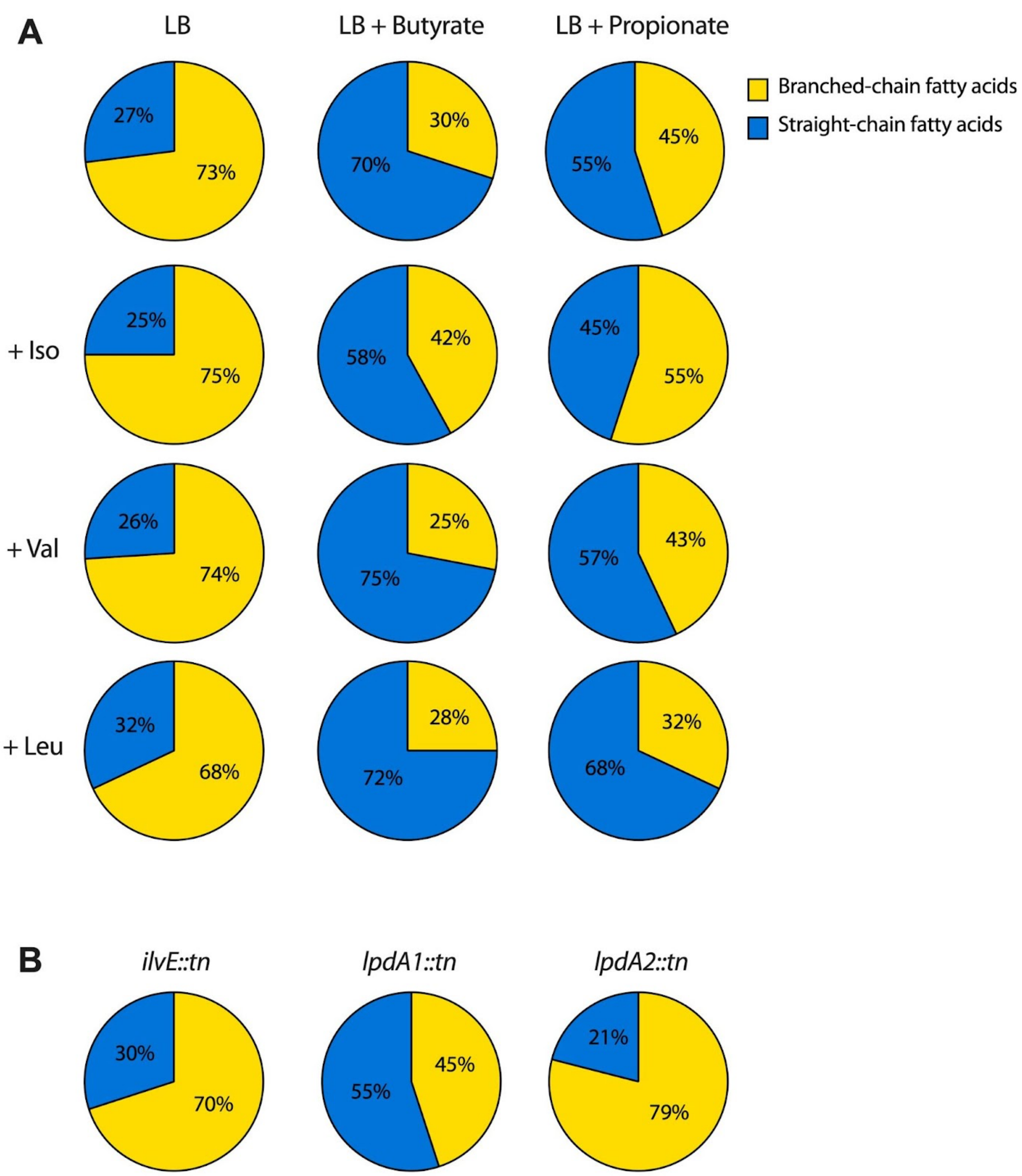

Figure S5. SCFAs alter *S. aureus* membrane lipid composition. (A) Ratio of branched-chain fatty acids to straight-chain fatty acids in *S. aureus* JE2 grown in LB with and without supplementation of sodium propionate or sodium butyrate, with or without supplementation of 1mg/mL BCAAs isoleucine (iso), valine (val), or leucine (leu). **(B)** ratio of branched-chain fatty acids to straight-chain fatty acids in *S. aureus* JE2 transposon mutants from the Nebraska Transposon Mutant Library.

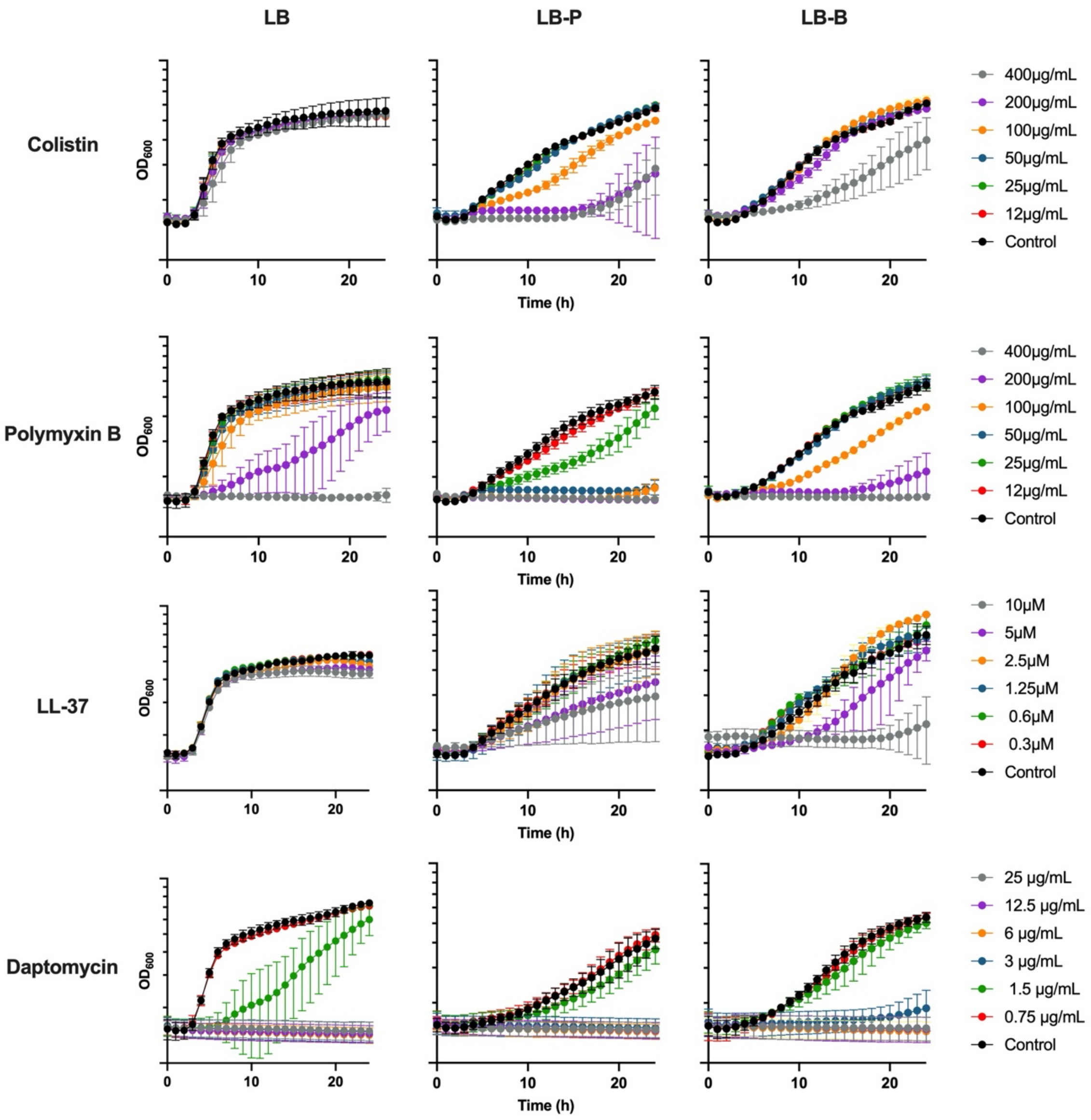
LB
LB-P
LB-B
Colistin
Polymyxin B
LL-37
Daptomycin
OD600
Time (h)
0
10
20
400μg/mL
200μg/mL
100μg/mL
50μg/mL
25μg/mL
12μg/mL
Control
10μM
5μM
2.5μM
1.25μM
0.6μM
0.3μM
25 μg/mL
12.5 μg/mL
6 μg/mL
3 μg/mL
1.5 μg/mL
0.75 μg/mL

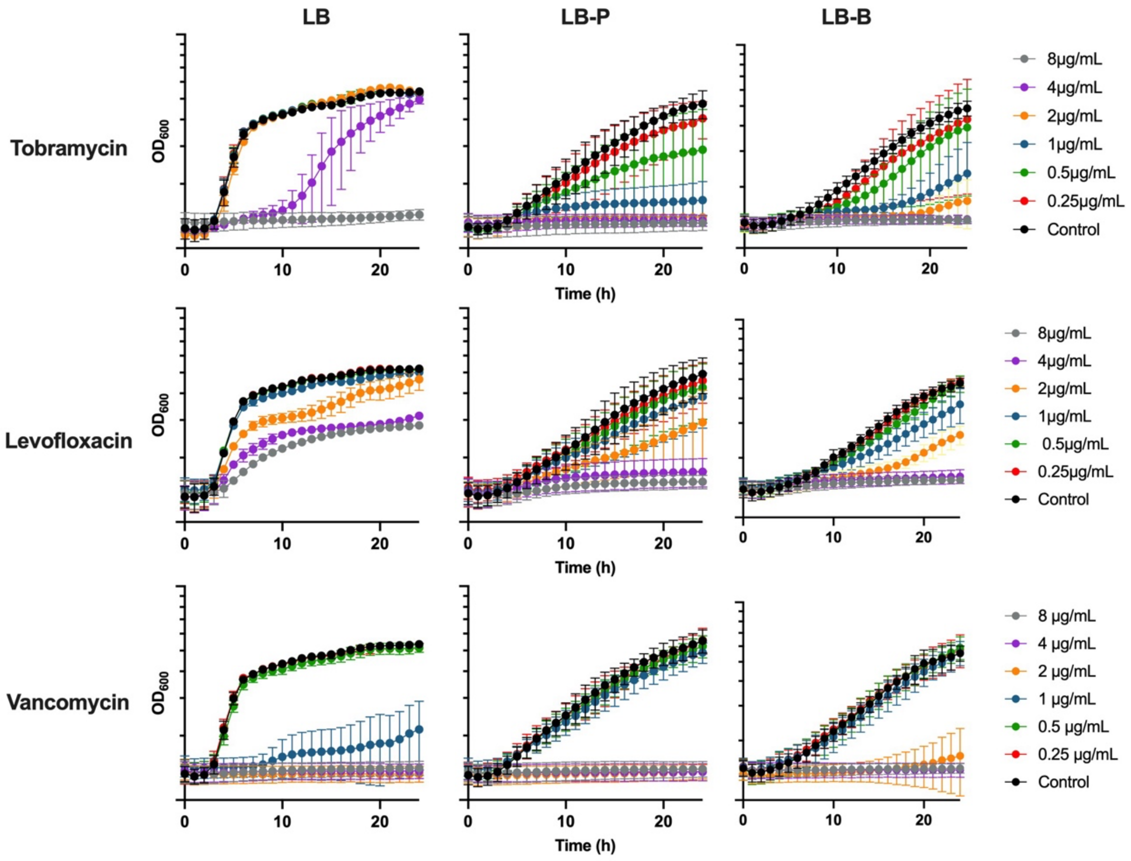

Figure S6. Raw growth curve data for the AUC heatmap shown in Figure 5a,b. Data represent growth of *S. aureus* JE2 grown LB +/- propionate or butyrate, +/- one antibiotic at increasing concentrations. n=4 growth curves per strain, per condition.

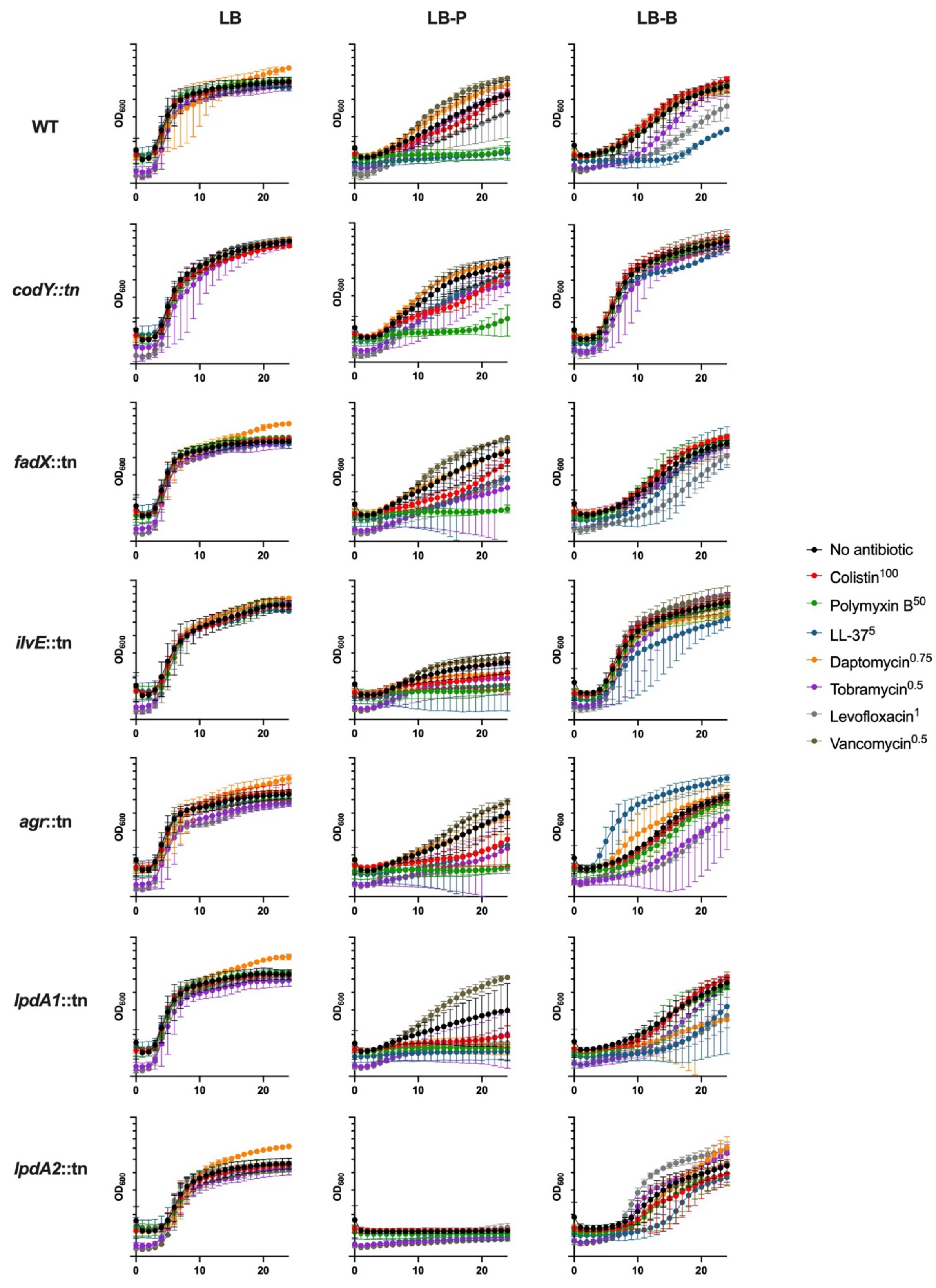

Figure S7. Raw growth curve data for the AUC heatmap shown in Figure 5c. Data represent growth of *S. aureus* JE2 and transposon mutants grown LB +/- propionate or butyrate, +/- one antibiotic at increasing concentrations. n=4 growth curves per strain, per condition.

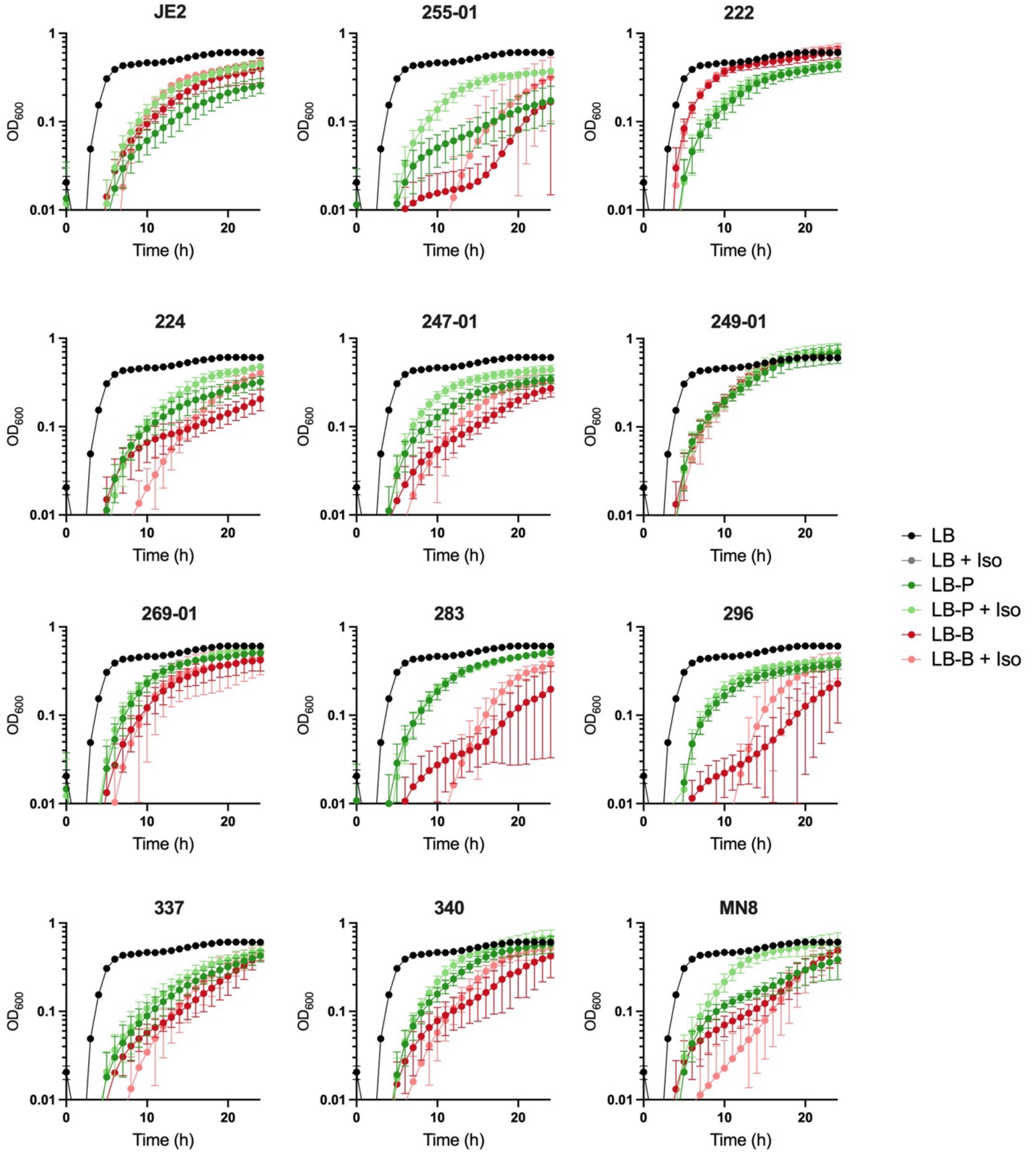

Figure S8. Raw growth curve data for the AUC heatmap shown in Figure 6a. Data represent growth of *S. aureus* JE2 and a panel of clinical isolates grown LB +/- propionate or butyrate, +/- BCAAs. n=3 growth curves per isolate, per condition.

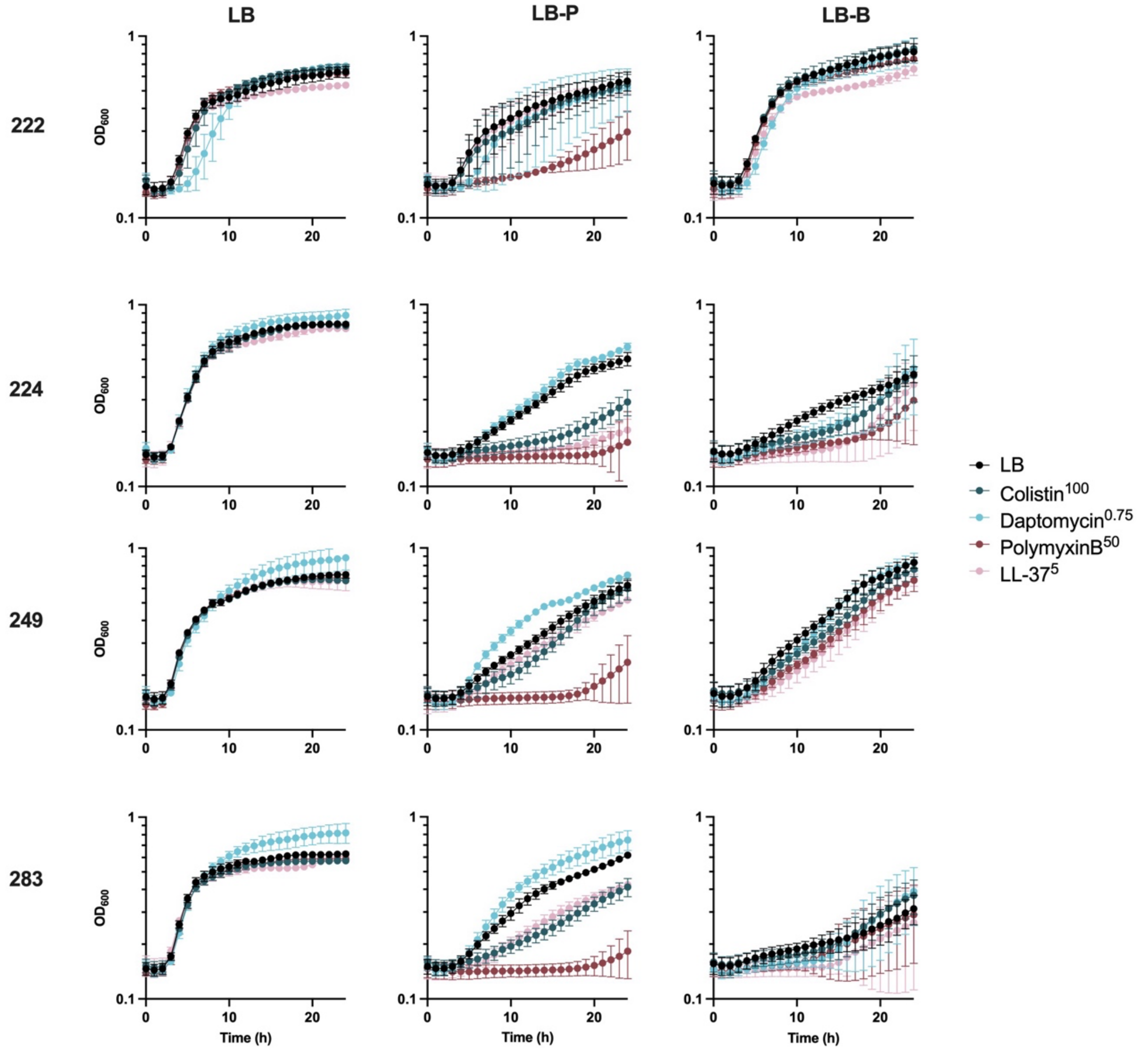

Figure S9. Raw growth curve data for the AUC heatmap shown in Figure 6c. Data represent growth of *S. aureus* JE2 and a panel of clinical isolates grown LB +/- propionate or butyrate, +/- one antibiotic. n=3 growth curves per strain, per condition.

A

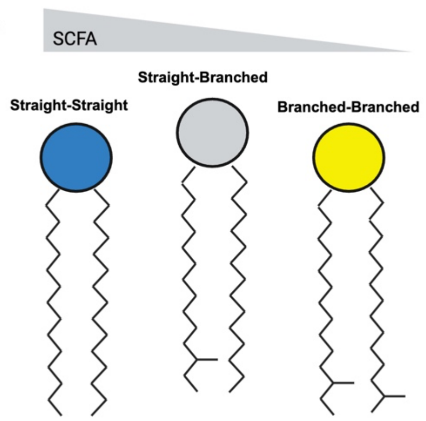

B

	LB	Propionate	Butyrate
**Membrane BCFA content**	73%	45%	30%
**+BCAA (1mg/mL)**			
isoleucine	75%	55%	42%
leucine	68%	32%	28%
valine	74%	43%	25%
**Membrane integrity**	Polarized	Compromised	Compromised
**Abx (~MIC, μg/mL)**			
polymixin B	200	50	100-200
colistin	> 400	200	400
daptomycin	3	1.5-3	3
vancomycin	1	2	2
levofloxacin	>8	4	2-4
tobramycin	8	1	1-2
LL-37 (μM)	>10	10	10
**Agr signaling (rel. P*agr*-mCherry fluorescence; Fletcher et al., 2022)**	1.0	0.351	0.104
**Competition with PA (rel. CFU)**	1.0	0.248	0.447
**Key mutant phenotypes**			
codY::tn	Rescues growth and membrane integrity on butyrate		
ilvE::tn	Rescues growth on butyrate, but further impairs growth and membrane integrity on propionate		

Fig. S10. (A) *S. aureus* membrane lipid composition shifts from predominately branched-chain fatty acids in the membrane to branched-straight or straight-straight isoforms during growth on propionate and butyrate. (B) Summary of the effects of short-chain fatty acids on lipid membrane composition and related phenotypes. *S. aureus* growth on propionate and butyrate leads to an increase in straight-chain fatty acids, decreased membrane polarization, increased sensitivity to membrane-targeting antibiotics and other classes of antimicrobials, impaired Agr signaling, and a competitive disadvantage against other airway pathogens (*e.g. P. aeruginosa)*. Many of these phenotypes are rescued by isoleucine, or by mutations in *codY,* encoding a master regulator, and *ilvE,* encoding a branched-chain amino acid transferase.

Dataset S1 (separate file). Nanostring data from *S. aureus* JE2 cultured in LB alone, and LB supplemented with sodium propionate and sodium butyrate

Dataset S2 (separate file). Proteomic data from *S. aureus* JE2 cultured in LB alone, and LB supplemented with sodium propionate and sodium butyrate

Dataset S3 (separate file). Comprehensive statistical analyses of each dataset.

SI References

1. P.D. Fey *et al.,* A genetic resource for rapid and comprehensive phenotype screening of nonessential *Staphylococcus aureus* genes. *mBio*, **4**, 10-1128 (2013)
2. D.A. Blomster-Hautamaa, P.M. Schlievert, P.M., “Preparation of toxic shock syndrome toxin-1 in Methods in enzymology vol. 165 (Academic Press, 1988) pp. 37-43).
3. L.G. Rahme *et al*., Common virulence factors for bacterial pathogenicity in plants and animals. Science, **268**, 1899-1902 (1995).
4. S.K. Lucas *et al.,* Anaerobic microbiota derived from the upper airways impact *Staphylococcus aureus* physiology. *Infect. Immun* **89**, 9 (2021).
5. M.N. Schroth, J.J. Cho, S.K. Green, S.D. Kominos, Microbiology Society Publishing, Epidemiology of *Pseudomonas aeruginosa* in agricultural areas. *J. Med. Microbiol.* **67,** 1191-1201 (2018).
6. C. D. Freeman *et al.*, Defective *pgsA* contributes to increased membrane fluidity and cell wall thickening in *Staphylococcus aureus* with high-level daptomycin resistance. *mSphere* **9,** e00115-24 (2024).
